# Histone‐lysine N‐methyltransferase *EHMT2* (G9a) inhibition mitigates tumorigenicity in Myc‐driven liver cancer

**DOI:** 10.1002/1878-0261.13417

**Published:** 2023-03-29

**Authors:** Dexter Kai Hao Thng, Lissa Hooi, Clarissa Chin Min Toh, Jhin Jieh Lim, Deepa Rajagopalan, Imran Qamar Charles Syariff, Zher Min Tan, Masturah Bte Mohd Abdul Rashid, Lei Zhou, Alfred Wei Chieh Kow, Glenn Kunnath Bonney, Brian Kim Poh Goh, Juinn Huar Kam, Sudhakar Jha, Yock Young Dan, Pierce Kah Hoe Chow, Tan Boon Toh, Edward Kai‐Hua Chow

**Affiliations:** ^1^ Cancer Science Institute of Singapore National University of Singapore Singapore Singapore; ^2^ NUS Centre for Cancer Research (N2CR), Yong Loo Lin School of Medicine National University of Singapore Singapore Singapore; ^3^ Department of Pharmacology, Yong Loo Lin School of Medicine National University of Singapore Singapore Singapore; ^4^ KYAN Therapeutics Singapore Singapore; ^5^ Department of Medicine, Yong Loo Lin School of Medicine National University of Singapore Singapore Singapore; ^6^ Division of Hepatobiliary & Pancreatic Surgery, Department of Surgery, University Surgical Cluster National University Health System Singapore Singapore; ^7^ Department of Hepatopancreatobiliary (HPB) and Transplant Surgery Singapore General Hospital and National Cancer Centre Singapore Singapore Singapore; ^8^ Department of Biochemistry, Yong Loo Lin School of Medicine National University of Singapore Singapore Singapore; ^9^ Department of Physiological Sciences, College of Veterinary Medicine Oklahoma State University Stillwater OK USA; ^10^ Academic Clinical Programme for Surgery Duke‐NUS Medical School Singapore Singapore; ^11^ The N.1 Institute for Health (N.1) National University of Singapore Singapore Singapore; ^12^ The Institute for Digital Medicine (WisDM), Yong Loo Lin School of Medicine National University of Singapore Singapore Singapore

**Keywords:** epigenetics, G9a, HCC, Myc

## Abstract

Hepatocellular carcinoma (HCC) is the third deadliest and sixth most common cancer in the world. Histone‐lysine N‐methyltransferase *EHMT2* (also known as G9a) is a histone methyltransferase frequently overexpressed in many cancer types, including HCC. We showed that Myc‐driven liver tumours have a unique H3K9 methylation pattern with corresponding G9a overexpression. This phenomenon of increased G9a was further observed in our c‐Myc‐positive HCC patient‐derived xenografts. More importantly, we showed that HCC patients with higher c‐Myc and G9a expression levels portend a poorer survival with lower median survival months. We demonstrated that c‐Myc interacts with G9a in HCC and cooperates to regulate c‐Myc‐dependent gene repression. In addition, G9a stabilises c‐Myc to promote cancer development, contributing to the growth and invasive capacity in HCC. Furthermore, combination therapy between G9a and synthetic‐lethal target of c‐Myc, CDK9, demonstrates strong efficacy in patient‐derived avatars of Myc‐driven HCC. Our work suggests that targeting G9a could prove to be a potential therapeutic avenue for Myc‐driven liver cancer. This will increase our understanding of the underlying epigenetic mechanisms of aggressive tumour initiation and lead to improved therapeutic and diagnostic options for Myc‐driven hepatic tumours.

## Graphical abstract

This study demonstrates that G9a and c‐Myc positively correlate and portend poorer survival in hepatocellular carcinoma (HCC). G9a is shown to interact with c‐Myc, regulating c‐Myc stability in HCC. G9a also plays a role in promoting cell proliferation and invasiveness of HCC. Importantly, synergy observed between G9a and CDK inhibitors mitigates organoid growth and tumour formation in HCC patient‐derived avatars.

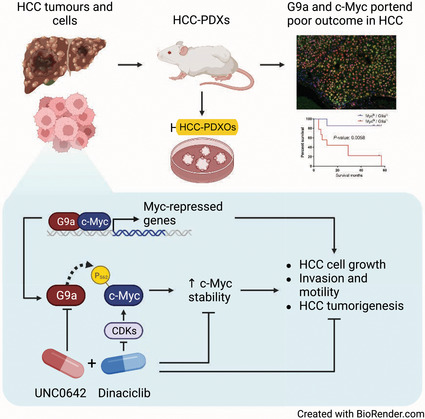

**Abbreviations**
ChIP: chromatin immunoprecipitation
CHX: cyclohexamide
EMT: epithelial–mesenchymal transition
FBS: foetal bovine serum
FIHC: fluorescence immunohistochemistry
FITC: fluorescein isothiocyanate
H3: histone 3
H3K27: histone 3 lysine 27
H3K9: histone 3 lysine 9
H3K9me2: histone 3 lysine 9 dimethylation
HCC: hepatocellular carcinoma
HCC‐PDX: hepatocellular carcinoma patient‐derived xenograft
HCC‐PDXO: hepatocellular carcinoma patient‐derived xenograft organoid
IACUC: Institutional Animal Care and Use Committee
IC_50_
: half‐maximal inhibitory concentration
OACD: orthogonal array composite design
PLA: proximity ligation assay
QPOP: quadratic phenotypic optimisation platform
qRT‐PCR: quantitative real‐time polymerase chain reaction
SB: sleeping beauty
SD: standard deviation

## 1 Introduction

Liver cancer is the third most common cause of cancer‐related death in the world with the highest incidence in Asia. Current treatments for HCC include surgical resection, liver transplantation and local ablation therapy. However, these treatments have limited efficacy in patients with advanced HCC. Currently, there are several FDA‐approved systemic therapies for advanced HCC patients, including immunotherapy‐based combination atezolizumab and bevacizumab, and multi‐kinase antagonists sorafenib, lenvatinib, regorafenib, cabozantinib and ramucirumab [1, 2, 3, 4, 5, 6]. However, these drugs improved progression‐free survival rates by only 3–7 months, with median overall survival being no longer than 2 years. This underscores the need to develop better therapeutics for the treatment of HCC.

Overexpression of c‐Myc is frequently observed in HCC [7]. c‐Myc, a potent oncogene commonly overexpressed in human cancers, plays an essential role in the regulation of cell growth, differentiation and apoptosis in both normal and neoplastic cells. More importantly, c‐Myc amplification portends a more aggressive HCC phenotype and poorer prognosis, suggesting a critical role in tumorigenesis [8, 9]. A key strategy for mitigating the oncogenic potential of c‐Myc is to target various synthetic lethal targets and vulnerabilities of c‐Myc [10]. Previous work in mouse embryonic stem cells has shown that c‐Myc plays a key role in the maintenance of mouse embryonic stem cells through epigenetic regulation by influencing the levels of histone 3 (H3) methylation [11]. However, the significance and mechanism of such epigenetic regulation by c‐Myc, and its value as a vulnerability of c‐Myc, has not been extensively explored in cancers.

G9a is a euchromatin‐localised histone methyltransferase that mediates the methylation of histone H3 at lysines 9 and 27 (H3K9 and H3K27) and is required for early embryogenesis [12]. Importantly, G9a has been shown to be overexpressed and correlated with poorer prognosis and malignancy in several solid tumours including HCC, head and neck squamous cell carcinoma, oesophageal squamous cell carcinoma, Ewing sarcoma, lung, breast, ovarian and colorectal cancers [13, 14, 15, 16, 17, 18, 19, 20]. These suggest the crucial role of G9a in the dysregulation of epigenetic pathways in tumorigenesis. c‐Myc has been found to interact with G9a protein in an IFI35/c‐Myc/Nmi complex that docks on the transcriptional repressive mark of H3K9 dimethylation (H3K9me2) [21]. In addition, chronic active G9a also stabilises c‐Myc within promoters of select genes to exert its transcriptional repression function in macrophages during endotoxin tolerance. Tu and colleagues demonstrated the interaction between c‐Myc and G9a in basal breast tumours whereby G9a inhibition suppresses c‐Myc‐dependent tumour growth, suggesting that G9a is an important epigenetic modulator of c‐Myc transcriptional repression [22]. Additionally, c‐Myc was recently shown to mediate the recruitment of G9a to the promoter of 15‐hydroxyprostaglandin dehydrogenase to silence its expression through enhanced H3K9 methylation in cholangiocarcinoma [23]. However, such regulation and interactions are not well described in the pathogenesis of HCC. In this study, we explore the underlying G9a‐mediated mechanisms of Myc‐driven aggressive hepatic tumour initiation as well as to identify potential therapeutic options for Myc‐driven hepatic tumours.

## 2 Materials and methods

### 2.1 HCC patient‐derived tumour xenografts (PDXs) and xenograft organoids (PDXOs)

HCC samples were obtained from patients at the National University Hospital, Singapore General Hospital and National Cancer Centre Singapore with written informed consent from 2015 to 2018. The collection of patient samples for generation and use of PDXs was performed under domain‐specific review board (DSRB) protocol and study approval by the Institutional Review Board of National University Hospital, Singapore (NHG‐DSRB Ref: 2011/01580), the SingHealth Central Institutional Review Board (CIRB Ref: 2016/2626 and 2018/2112) and human biomedical research protocol and study approval by the NUS‐Institutional Review Board (NUS‐IRB Ref: LH‐18‐037E). The study methodologies conformed to the standards set by the Declaration of Helsinki. Tissue samples were finely minced and mixed with Matrigel™ (1 : 1 ratio) before being grafted subcutaneously into the left flanks of 6‐ to 8‐week‐old female NOD.Cg‐Prkdc^severe combined immunodeficiency (scid)^Il2rg^tm1Wjl^/SzJ (NOD scid gamma [NSG]) mice from The Jackson Laboratory to establish the HCC‐PDXs. HCC‐PDXs were serially passaged and transplanted when the tumour volumes have reached the end point of 2000 mm^3^. All mice were maintained in an enriched environment on a 12 h light–dark cycle and fed with Teklad global 18% protein rodent diet. All animal studies were performed in accordance with guidelines and protocols (R18‐0907) approved by the National University of Singapore (NUS) Institutional Animal Care and Use Committee (IACUC).

Organoid cultures were established from HCC‐PDXs as described previously [24, 25]. Briefly, extracted HCC‐PDXs were minced into fine pieces and incubated with 1 mg·mL^−1^ collagenase/dispase® for 30 min. Digested tumour pieces were passed through a 100 μm cell strainer and centrifuged. Isolated tumour cells were resuspended in DMEM/F‐12 and Matrigel™ (1 : 4 ratio) for seeding, and cultured in DMEM/F‐12 supplemented with necessary growth factors as described previously [26].

### 2.2 Quadratic phenotypic optimisation platform (QPOP)

An eight drug‐three dosages (IC_0_, IC_15_, IC_30_) QPOP was performed in this study. The drug panel included UNC0642 (G9a inhibitor), sorafenib and regorafenib (HCC standard of care), dinaciclib (CDK1 and CDK9 inhibitor), FDA‐approved oncology drugs omipalisib (PI3K/mTOR inhibitor), pacritinib (JAK2 inhibitor) and carfilzomib and ixazomib (proteasome inhibitors) [27, 28]. HCC‐PDXOs were screened against log dose concentrations of the panel of eight drugs for 72 h to establish their respective dose–response curves and respective half‐maximal inhibitory concentrations (IC_50_). IC_15_ and IC_30_ concentrations were intrapolated from the dose–response curves. An orthogonal array composite design (OACD) was used to determine the 91 combinations necessary for sufficient factor screening and in‐depth analyses of the HCC‐PDXO responses to the eight drugs at three dosages [29]. The Mini Janus robotic liquid handler was used to prepare the 91 combinations and was screened against the HCC‐PDXOs for 72 h. The viability of HCC‐PDXOs was then fitted into a second‐order quadratic series for QPOP analyses as described previously [24].

### 2.3 Cell culture and drug treatment

Human HCC cell lines (Hep3B (RRID:CVCL_0326), SNU387 (RRID:CVCL_0250), SNU398 (RRID:CVCL_0077) and SNU449 (RRID:CVCL_0454)), and murine liver cell line AML‐12 (RRID:CVCL_0140) were obtained from American Type Cell Culture (ATCC). Human HCC cell lines BEL7402 (RRID:CVCL_5492) and Huh7 (RRID:CVCL_0336) were kind gifts from Dr Polly Leilei Chen (Cancer Science Institute of Singapore), and HCCLM3 (RRID:CVCL_6832) was a kind gift from Dr Masha Babak (City University of Hong Kong). LT2‐Myc cells from the murine hepatoblastoma tumour model were obtained from Dr J. Michael Bishop (University of California, San Francisco) as described previously [30, 31]. All cell lines have been authenticated within the last 3 years via the ATCC short tandem repeat profiling service. LT2‐Myc, BEL7402, LM3, Hep3B and Huh7 were grown in DMEM supplemented with 10% foetal bovine serum (FBS), while SNU387, SNU398 and SNU449 cells were grown in RPMI with 10% FBS. AML‐12 was cultured in DMEM supplemented with 10% FBS, insulin, transferrin, selenium and dexamethasone as per ATCC's instructions. All cell lines were grown in a humidified atmosphere containing 5% CO_2_ at 37 °C. All experiments were performed with mycoplasma‐free cells.

### 2.4 Hydrodynamic transfection mouse model

To generate c‐Myc, Akt/Ras and Akt/β‐catenin hepatic tumour mouse models, respectively, 20 μg of plasmids encoding for c‐Myc or 20 μg of two plasmids encoding for myristoylated Akt (myr‐Akt) and oncogenic N‐Ras (NRASV12) or 20 μg of two plasmids encoding for myr‐Akt and Δ90Nβ‐catenin was mixed with 2 μg of plasmids encoding the Sleeping Beauty (SB) transposase in 2.0 mL of phosphate‐buffered saline and injected into the lateral tail vein of 6‐ to 8‐week‐old female wild‐type FVB/N mice from InVivos [31, 32, 33]. All mice were maintained in an enriched environment on a 12 h light–dark cycle and fed with Teklad global 18% protein rodent diet. All animal studies were performed in accordance with guidelines and protocols (R18‐1508) approved by the NUS IACUC.

### 2.5 *In vivo* drug treatment

For subcutaneous xenograft implantation for drug treatment, 1 × 10^6^ dissociated HCC‐PDX cells were mixed with 100 μL of Matrigel™ (1 : 1 ratio) before subcutaneously implanted into the left flanks of 6‐ to 8‐week‐old female NOD.Cg‐Prkdc^severe combined immunodeficiency (scid)^Il2rg^tm1Wjl^/SzJ (NOD scid gamma [NSG]) mice from The Jackson Laboratory. Tumours were allowed to grow to approximately 100 mm^3^ before randomised into their respective treatment groups: vehicle intraperitoneally thrice a week (*n* = 5), sorafenib 30 mg·kg^−1^ thrice a week by oral gavage (*n* = 4), UNC0642 5 mg·kg^−1^ intraperitoneally daily (*n* = 5), dinaciclib 5 mg·kg^−1^ intraperitoneally thrice a week (*n* = 5), UNC0642 + dinaciclib intraperitoneally (*n* = 4). Tumour‐bearing mice were treated up to 42 days or when the tumour has reached the end point of 2000 mm^3^. All mice were maintained in an enriched environment on a 12 h light–dark cycle and fed with Teklad global 18% protein rodent diet. All animal studies were performed in accordance with guidelines and protocols (R18‐0907) approved by the NUS IACUC.

### 2.6 Lentiviral‐ and siRNA‐mediated knockdown

Lentiviral‐compatible shRNA clones were obtained from the Open Biosystems (Huntsville, AL, USA) pLKO.1 shRNA library against *EHMT2* (TRCN0000115667 [sh*EHMT2*‐1] and TRCN0000115671 [sh*EHMT2*‐2]). Lentiviral particles were generated by co‐transfecting shRNAs into HEK293T cells with third‐generation lentiviral plasmids using Fugene® 6 Transfection Reagent (Promega, Madison, WI, USA). Cells were transduced with the shRNA‐bearing lentiviral particles, and the knockdown efficiency was determined by immunoblot analysis. G9a‐depleted stable cells were selected and maintained in culture with 2 μg·mL^−1^ puromycin.

SMARTpool ON‐TARGETplus™ small interfering RNA (siRNAs) targeting *MYC* transcripts were obtained from Dharmacon (Lafayette, CO, USA) and reconstituted according to the manufacturer's protocol. BEL7402 cells were transiently transfected with 50 nm of *MYC* siRNAs with Lipofectamine RNAiMAX following the manufacturer's instructions. Knockdown efficiency was determined by quantitative real‐time polymerase chain reaction.

### 2.7 Immunoblot analysis

Cells were lysed in Pierce® RIPA buffer containing protease, and phosphatase inhibitor cocktail tablets (Roche, Mannheim, Germany). Equal amounts of protein lysate were resolved by SDS/PAGE and electrotransferred onto PVDF membranes. Membranes were processed according to standard procedure and proteins were detected using the ChemiDoc™ MP imaging system (Bio‐Rad, Hercules, CA, USA). Antibodies used for immunoblot analyses included antibodies for c‐Myc (Abcam, Cambridge, UK), G9a (Abcam), p‐c‐Myc (S62) (Cell Signaling Technology, Danvers, MA, USA), p‐c‐Myc (T58) (Cell Signaling Technology), H3K9me1 (Cell Signaling Technology), H3K9me2 (Cell Signaling Technology), H3K9me3 (Cell Signaling Technology), H3 (Cell Signaling Technology), Sox9 (Cell Signaling Technology), active β‐catenin (Merck Millipore, Temecula, CA, USA), total β‐catenin (BD Transduction, San Jose, CA, USA), p62 (Cell Signaling Technology), LC3B (Cell Signaling Technology), cleaved caspase 3 (Cell Signaling Technology), caspase 3 (Cell Signaling Technology), PARP (Cell Signaling Technology) and β‐actin (Sigma).

### 2.8 Quantitative real‐time polymerase chain reaction (RT‐PCR)

RNA was isolated using the RNeasy Plus kits (Qiagen) and reverse transcribed into cDNA using the iScript Reverse Transcription Supermix (Bio‐Rad Laboratories) according to the manufacturers' instructions. Quantitative RT‐PCR was done in triplicates and subjected to 40 amplification cycles of PCR (QuantStudio™ 3 Real‐Time PCR System). See Table S1 for primers used in this study.

### 2.9 Immunohistochemistry and tissue microarray analysis

Tissues were fixed in 4% paraformaldehyde immediately after isolation and embedded in paraffin. Tissue sections of 4 μm thickness were stained for immunohistochemistry. Ten HCC‐PDX tissues and a tissue microarray (TMA) of 77 human liver cancer patients (OutdoBiotech, Shanghai, China) were serially stained using the Opal workflow (Akoya Biosciences, Marlborough, MA, USA) with c‐Myc primary antibody (1 : 200, Abcam) for 1 h followed by HRP‐conjugated secondary antibody and tyramide signal amplification (TSA) by fluorescein isothiocyanate (FITC). Tissues were subsequently stained with G9a primary antibody (Abcam), and the same TSA system was applied using Cyanine 5 (Cy5) as the fluorescent stain. DAPI was used as the nuclear counterstain. Image acquisition and signal quantification analysis of the tissues were performed using the Vectra imaging system (Akoya Biosciences) and inForm software (Akoya Biosciences) respectively. Tissues were categorised into low and high expression using the first and third quartile mean intensities as thresholds respectively.

### 2.10 Immunofluorescence

HCC‐PDXOs were seeded in 96‐well plates in 80% Matrigel for 24 h prior to UNC0642 and dinaciclib treatment for 48 h. Treated organoids were fixed in 4% paraformaldehyde prior to permeabilisation with 0.5% Triton X‐100. Blocking buffer (10% horse serum, 0.15 bovine serum albumin, 0.2% Triton X‐100) was added to the organoids for 2 h prior to incubation with primary antibodies against LC3B (Cell Signaling Technology) overnight. Organoids were then washed and incubated in secondary antibodies and stained with DAPI for 2 h before imaging using the confocal laser scanning microscope (LSM880 Airy Scan, Carl Zeiss, Oberkochen, Germany).

### 2.11 Proximity ligation assay (PLA)

2 × 10^4^ cells were seeded in chamber slides 24 h prior to fixation with 4% paraformaldehyde for 30 min. Cells were subsequently permeabilised with 0.1% Triton‐X for 2 min. PLA was performed using the Duolink® PLA mouse/rabbit kit (Sigma) following the manufacturer's instructions. Briefly, cells were blocked and incubated with primary antibodies for c‐Myc (Merck Millipore) and G9a (R&D Systems, Minneapolis, MN, USA) overnight at 4 °C. Oligomer‐conjugated probes were added for 30 min at 37 °C prior to the addition of polymerase for rolling circle amplification for 100 min. Slides were mounted with Duolink® *In Situ* Mounting Media with DAPI. Cells were then imaged with Zeiss Imager M2 and analysed using imagej (National Institutes of Health, Bethesda, MD, USA).

### 2.12 Chromatin immunoprecipitation (ChIP) assay

ChIP experiments against c‐Myc antibody (Abcam) and G9a antibody (Abcam) were performed on BEL7402 cells. Cells were fixed in 1% paraformaldehyde for 10 min and then quenched with 0.25 m glycine. Cells were lysed with SDS lysis buffer, sonicated to 200–500 bp fragments for 20 cycles, and incubated with respective c‐Myc and G9a antibodies overnight. The antibody complexes were linked to pre‐equlibrated Protein G Sepharose® beads (Abcam) and washed thoroughly before elution in fresh elution buffer (0.1 m NaHCO3, 10% SDS) with agitation. Eluates were incubated in 200 mm NaCl overnight at 65 °C, followed by proteinase K digestion. Chromatin immunoprecipitated DNA was purified using Qiaquick PCR Purification Kit (Qiagen), followed by quantitative real‐time polymerase chain reaction (qRT‐PCR) using primers described in Table S1.

### 2.13 Cycloheximide protein synthesis inhibition assay

BEL7402 cells were seeded for 24 h and treated with UNC0642 (Tocris, Bristol, UK) at 10 μm for 24 h. G9a‐depleted BEL7402 cells were seeded for 24 h. Drug‐treated or G9a‐depleted cells were then treated with 100 μg·mL^−1^ cycloheximide (CHX; Sigma‐Aldrich) and harvested at indicated time points using RIPA buffer. Cells were lysed and subjected to immunoblot analysis as described above. Band intensities were determined using the image lab™ software (Bio‐Rad).

### 2.14 Wound healing and AIM biotech invasion assays

3 × 10^5^ BEL7402 cells were seeded per well and grown to 90–95% confluency. A p10 tip was used to scratch the cell monolayer and incubated with serum‐free medium at 37 °C. Brightfield images were taken over 48 h and analysed with imagej (National Institutes of Health). For invasion assay, Matrigel was introduced into the middle channel of AIM Biotech chip and was left to solidify at 37 °C for 15 min. BEL7402 cells in serum‐free media were seeded at a density of 4 × 10^6^ cells·mL^−1^ in the channel on the left, while media containing 10% FBS was introduced on the right. Cells were incubated at 37 °C and media was replaced daily. At indicated time points, cells were imaged with Operetta® High‐Content Imaging System and analysed with imagej (National Institutes of Health).

### 2.15 Statistical analysis

All experimental data were at least performed in triplicates (unless otherwise stated), the results averaged and the SD or standard error of the mean (SEM) calculated. All statistical tests were performed on graphpad prism 8 software (GraphPad, San Diego, CA, USA). *P* < 0.05 was accepted as statistically significant.

## 3 Results

### 3.1 G9a and c‐Myc positively correlate and portend poorer survival in HCC

**Fig. 1:**
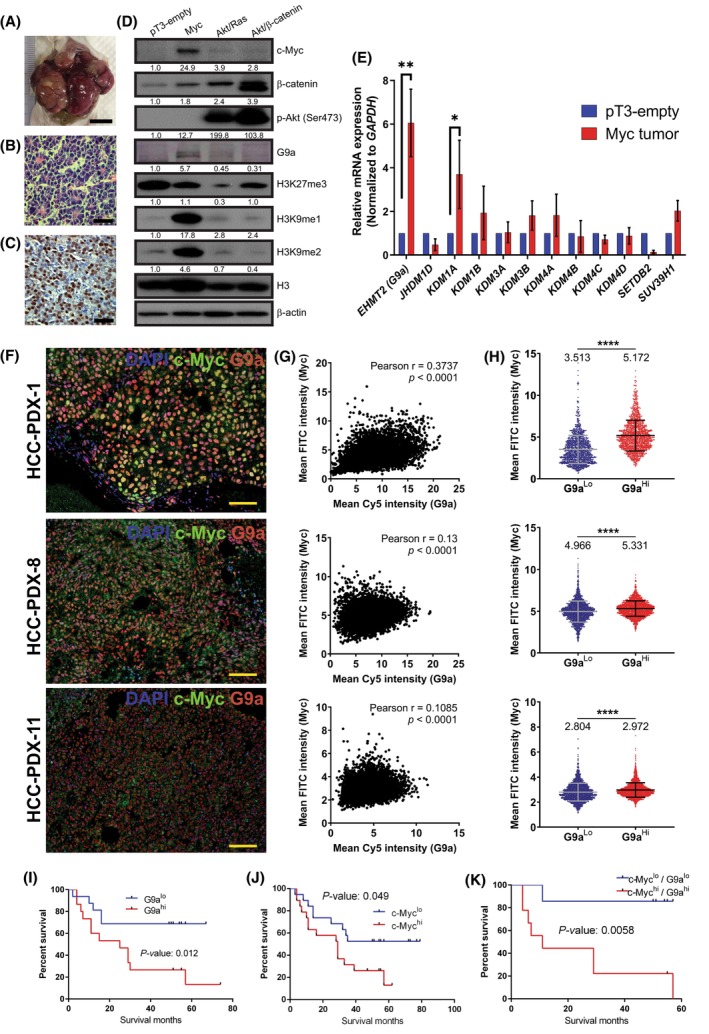
G9a and c‐Myc are positively correlated and portend poorer overall survival in hepatocellular carcinoma (HCC). (A) Gross image of Myc‐driven hepatic tumour induced by hydrodynamic transfection (*n* = 3). Scale bar denotes 1 cm. (B) Haematoxylin and eosin (H&E) staining of Myc‐driven hepatic tumour (*n* = 3). Scale bar denotes 50 μm. (C) c‐Myc expressing cells in Myc‐driven hepatic tumour (*n* = 3). Scale bar denotes 50 μm. (D) Histone 3 lysine 9 (H3K9) methylation and G9a are upregulated in Myc‐driven hepatic tumours as compared to other oncogene‐driven hepatic tumours such as Akt/β‐catenin and Akt/Ras. β‐Actin was used as the loading control. Densitometry analyses for all proteins and histone marks were normalised to β‐Actin and total histone 3 (H3) respectively. Densitometry analyses were represented as means below the immunoblots (*n* = 2). (E) Real‐time polymerase chain reaction analysis showing mRNA expression of histone methyltransferases that mediate H3K9 monomethylation and H3K9 dimethylation. Genes were normalised to housekeeping gene, *GAPDH*. Data represented as mean ± SD (*n* = 3). *P*‐values were determined by Student's *t*‐test. *P < 0.05; **P < 0.01. (F) Multi‐spectral imaging showing immunofluorescence co‐staining of DAPI (blue), c‐Myc (green) and G9a (red) in three HCC‐patient‐derived xenograft (PDX) tissues (*n* = 5). Scale bar denotes 100 μm. (G) Scatter plots of G9a and c‐Myc intensity in three HCC‐PDX tissues showing positive correlation [*n* = 5432 cells (PDX‐1), 7798 cells (PDX‐8), 9359 cells (PDX‐11)]. (H) Staining intensity of c‐Myc in G9a^hi^ and G9a^lo^ cells. Data represented as mean ± SD [*n* = 1358 cells (PDX‐1), 1949 cells (PDX‐8), 2339 cells (PDX‐11)]. *P*‐values were determined by Student's *t*‐test. ****P < 0.0001. (I) Kaplan–Meier plot of overall survival of HCC patients stratified by G9a protein expression, *n* = 16 (G9a^lo^), 15 (G9a^hi^). (J) Kaplan–Meier plot of overall survival of HCC patients stratified by c‐Myc protein expression, *n* = 19 (c‐Myc^lo^), 19 (c‐Myc^hi^). (K) Kaplan–Meier plot of overall survival of HCC patients stratified by both G9a and c‐Myc co‐expression, *n* = 7 (c‐Myc^lo^/ G9a^lo^), 9 (c‐Myc^hi^/G9a^hi^). Survival analyses were performed using log rank (Mantel–Cox) test.

We first examined G9a levels in a Myc‐driven hepatic tumour mouse model using sleeping beauty (SB) transposon hydrodynamic transfection system (Fig. 1A–C). Western blot analysis showed that the Myc tumour has a unique high expression of H3K9 mono‐ and di‐methylation signature as compared to the other oncogene‐driven tumours such as the Akt/Ras and Akt/β‐catenin tumours and the pT3‐empty control (Fig. 1D). In addition, we also observed high G9a expression only in the Myc‐driven tumours (Fig. 1D), suggesting that the high global H3K9 mono‐ and di‐methylation signature is mediated by G9a, the key histone methyltransferase that catalyses this modification. Furthermore, qRT‐PCR analysis of major histone methyltransferases that mediate H3K9 dimethylation demonstrated that *EHMT2* is highly expressed in the Myc‐driven hepatic tumour compared to the pT3‐empty control (Fig. 1E). Collectively, these data suggest that G9a is highly expressed in Myc‐driven hepatic tumours.

To demonstrate the clinical relevance of studying c‐Myc and G9a expression in HCC, we proceeded to examine the G9a and c‐Myc transcript levels and protein expression levels in a panel of 10 HCC‐PDXs by qRT‐PCR and multispectral quantitative fluorescent immunohistochemical (FIHC) staining respectively. mRNA expression levels of *EHMT2* and *MYC* did not exhibit a significant correlation (Fig. S1A). Using FIHC, we were able to reliably quantitate the nuclear expression of both c‐Myc and G9a protein within a single cell in their native tissue microenvironment (Fig. 1F, Fig. S1B). Our results showed that in a sample of 10 HCC‐PDXs, c‐Myc and G9a were largely positively correlated at the protein expression level in a subset of the HCC‐PDXs (Fig. 1G, Fig. S1C,D). Next, we stratified the G9a mean intensity into two distinct cohorts (G9a^lo^‐lower 25 percentile and G9a^hi^‐upper 25 percentile) and determined the percentage of c‐Myc‐positive cells in these two groups. We observed that collectively, the G9a^hi^ group contains significantly higher proportions of c‐Myc‐positive cells compared to the G9a^lo^ group across the cells of all 10 PDXs (Fig. S1E) and in most of the individual PDXs (Fig. 1H, Fig. S1F). These data collectively establish a correlation between c‐Myc and G9a in liver cancer and suggest that the interaction between G9a and c‐Myc is primarily at the protein level as opposed to co‐expression of either gene.

To determine the prognostic and clinical significance of G9a and c‐Myc in HCC, we examined the protein expression levels of G9a and c‐Myc in 77 HCC patient tumour specimens with matched normal adjacent tissues and known clinical follow‐up records using multispectral quantitative FIHC platform. We stratified the G9a mean intensities of these patient specimens into G9a^hi^ (upper 25 percentile) and G9a^lo^ (lower 25 percentile) cohorts and showed that patients with high expression of G9a (G9a^hi^) portend a poorer overall survival when compared with those of lower G9a expressions (G9a^lo^; Fig. 1I). We also analysed the c‐Myc expression levels in the same group of patients, divided them into their respective c‐Myc^hi^ (upper 25 percentile) and c‐Myc^lo^ (lower 25 percentile) cohorts, and showed that patients in the c‐Myc^hi^ cohort portend a poorer survival than patients in the c‐Myc^lo^ group as previously described (Fig. 1J). More importantly, when we analysed HCC patients that co‐expressed both high c‐Myc and G9a, we could demonstrate a higher probability in terms of predicting overall survival than individual gene expression (Fig. 1K), suggesting that both c‐Myc and G9a plays important roles in HCC patient survival and could be used together as potential prognostic markers.

### 3.2 Myc interacts with G9a in HCC

**Fig. 2:**
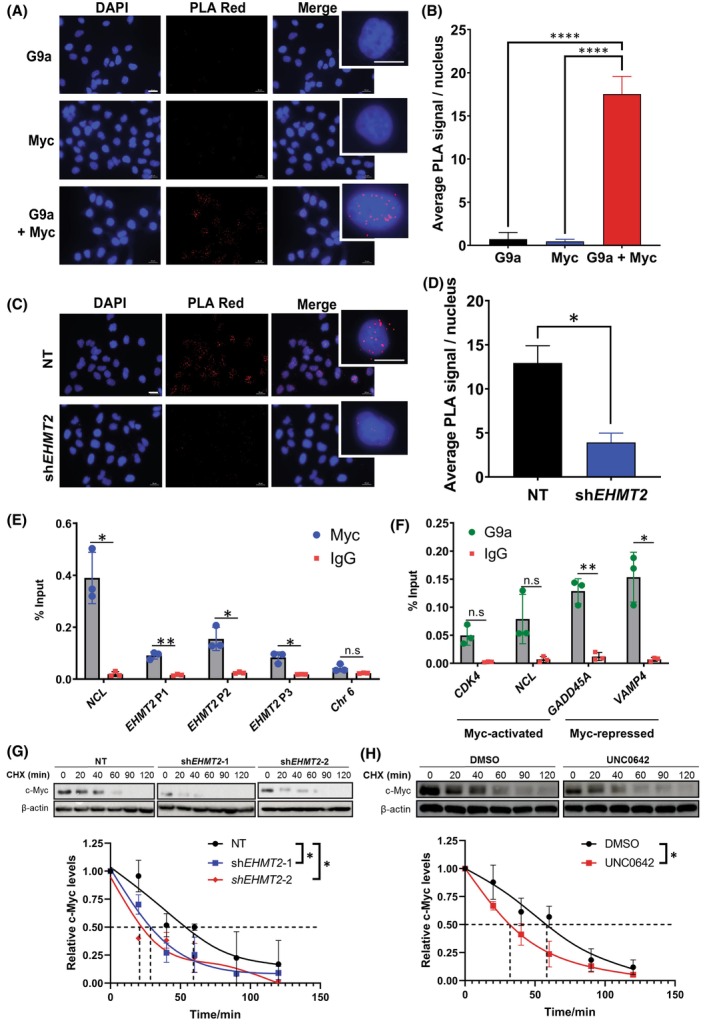
Myc interacts with G9a in hepatocellular carcinoma (HCC). (A) Proximity ligation assay (PLA) of single antibody controls or G9a and Myc together in BEL7402 cells. Representative immunofluorescent images of DAPI nuclear staining, positive PLA signal (Texas Red) and merged images (*n* = 3). Scale bar denotes 20 μm. Enlarged images are single representative nuclei selected from respective merged images. (B) Quantification of average positive PLA signal per nucleus from three independent biological repeats. Data represented as mean ± SD (*n* = 3). *P*‐values were determined by one‐way ANOVA. ****P < 0.0001. (C) PLA of G9a and Myc together on G9a‐depleted BEL7402 cells (sh*EHMT2*) and the non‐targeting control (NT). Representative immunofluorescent images of DAPI nuclear staining, positive PLA signal (Texas Red) and merged images (*n* = 3). Scale bar denotes 20 μm. Enlarged image is a single representative nucleus selected from the merged image. (D) Quantification of average positive PLA signal per nucleus from three independent repeats. Data represented as mean ± SD (*n* = 3). *P*‐values were determined by Student's *t*‐test. *P < 0.05. (E) Real‐time polymerase chain reaction (RT‐PCR) analysis of *EHMT2* promoter regions following chromatin immunoprecipitation (ChIP) assay on BEL7402 cells using c‐Myc antibody. Positive control: *NCL*; Negative control: Chr6. Data represented as mean ± SD of % input (*n* = 3). *P*‐values were determined by Student's *t*‐test. *P < 0.05; **P < 0.01; n.s, no significance. (F) G9a ChIP‐RT‐PCR in BEL7402 at both Myc‐activated (*CDK4, NCL*) and Myc‐repressed genes (*GADD45A* and *VAMP4*) with IgG as the isotype control. Data represented as mean ± SD of % input (*n* = 3). *P*‐values were determined by Student's *t*‐test. *P < 0.05; **P < 0.01; n.s., no significance. (G) Stability of c‐Myc protein was analysed by immunoblot analysis of whole cell lysates prepared from BEL7402 cells transduced with NT and two independent shRNAs against *EHMT2*. Protein expression was measured over a time course of 120 min after adding cycloheximide (CHX). Data represented as mean ± SD (*n* = 3). *P*‐values were determined by paired *t*‐test. *P < 0.05. (H) CHX assay on BEL7402 cells treated with vehicle or UNC0642 for 24 h. β‐Actin was used as the loading control. The reading of c‐Myc density was normalised to the reading of β‐actin density. Data represented as mean ± SD (*n* = 3). p‐values were determined by paired *t*‐test. *P < 0.05. Dashed lines represent the corresponding half‐lives of c‐Myc where relative c‐Myc abundance is at 0.5.

Our results have shown that the protein expression levels of c‐Myc and G9a were closely correlated in a panel of HCC‐PDX samples. We then performed proximity ligation assay (PLA) in BEL7402 HCC cell line to determine the *in situ* interactions between G9a and c‐Myc. A significantly higher positive fluorescence signal was observed in cell nuclei with both c‐Myc and G9a antibodies compared to the single antibody controls (Fig. 2A,B), suggesting that endogenous G9a colocalises with c‐Myc within an interacting distance of 40 nm. To validate the specificity of PLA, we depleted G9a in BEL7402 and compared the average positive PLA signals to its NT control. Importantly, we observed a significant reduction in the PLA signals in G9a‐depleted BEL7402 (Fig. 2C,D), validating the specificity of the PLA.

Next, to investigate if c‐Myc transcription factor occupies and regulates the expression of G9a, chromatin immunoprecipitation (ChIP) analysis was performed with anti‐Myc antibody in BEL7402 cells. Three representative regions spanning approximately 100–500 base pairs upstream of the transcription initiation start site of the *EHMT2* (G9a) gene were investigated (Fig. S2A). Our results showed that c‐Myc occupies the region of DNA fragment at the *EHMT2* promoter at all three regions indicated as ‘P1’, ‘P2’ and ‘P3’ with a significant enrichment as compared to its positive control nucleolin (*NCL*) (Fig. 2E) [34]. Notably, however, depletion of c‐Myc via small interfering RNA did not reduce *EHMT2* expression (Fig. S2B). These results demonstrate that while c‐Myc directly binds to the promoter of *EHMT2*, its role at the promoter is not clearly defined.

We next explored G9a‐Myc interaction at the chromatin level using ChIP‐qRT‐PCR for G9a at cyclin‐dependent kinase 4 (*CDK4*) and *NCL* which are Myc‐activated genes, as well as growth arrest and DNA damage‐inducible alpha (*GADD45A*) and vesicle‐associated membrane protein 4 (*VAMP4*) which are Myc‐repressed genes [22]. We observed significant binding of G9a at only Myc‐repressed genes but not Myc‐activated genes (Fig. 2F). This suggests a role for G9a in regulating Myc‐repressed genes. Binding of G9a at Myc‐activated genes albeit at insignificant levels could be attributed to stochastic binding of G9a to c‐Myc due to its structural compatibility as opposed to its enzymatic function. For further validation of G9a as a co‐repressor partner of c‐Myc in mediating transcriptional repression of Myc‐repressed genes, we conducted a G9a ChIP‐qRT‐PCR in G9a‐depleted BEL7402 cells. Importantly, we showed a significant decrease in binding at the promoters of only Myc‐repressed genes (*GADD45A* and *VAMP4*) in G9a‐depleted BEL7402 cells when compared with the NT (Fig. S2C). This further highlights the finding that G9a has a co‐repressive role in facilitating c‐Myc‐dependent transcriptional repression of c‐Myc target genes, and not in co‐activating Myc‐actived genes.

### 3.3 G9a inhibition reduces c‐Myc stability in HCC cells

We proceeded to investigate the role of G9a in regulating c‐Myc at the transcript and protein level. Our qRT‐PCR results showed that *MYC* mRNA was elevated in G9a‐depleted cells (Fig. S2D). Acute treatment with small molecule G9a inhibitor, UNC0642, however, suppressed *MYC* transcript levels (Fig. S2E), suggesting that while G9a enzymatically promotes *MYC* transcription, its role in regulating *MYC* expression is dispensable in G9a‐depleted contexts.

We next determined the degradation rate of the c‐Myc protein in G9a‐depleted BEL7402 cells and examined the c‐Myc protein expression along a time course after adding a protein synthesis inhibitor, cycloheximide (CHX). We observed that the G9a‐depleted cells had a more rapid degradation rate of the c‐Myc protein (faster c‐Myc decay) following inhibition of protein synthesis as compared to the NT control (Fig. 2G). We also compared c‐Myc decay rate in UNC0642‐treated cells and obtained similar results (Fig. 2H). As post‐translational dephosphorylation of c‐Myc at serine‐62 (S62) and phosphorylation of threonine‐58 (T58) have been demonstrated to destabilise c‐Myc protein, we went on to determine the phosphorylation status of c‐Myc T58 and S62 in the BEL7402 cells [35, 36, 37]. Immunoblot analyses demonstrated that both depletion and inhibition of G9a result in a significant reduction of the oncogenic phosphorylated S62 residue on c‐Myc, priming c‐Myc for polyubiquitylation and degradation (Fig. S2F) [38]. A reduction in T58 phosphorylation in c‐Myc was also observed in G9a‐depleted cells as T58 phosphorylation was contingent on S62 phosphorylation (Fig. S2F) [39]. These data suggest that G9a maintains the stability of c‐Myc protein in HCC cells by preserving phosphorylation at S62 position of c‐Myc.

### 3.4 Inhibition of G9a reduces proliferation and c‐Myc levels in HCC cells with high endogenous G9a

**Fig. 3:**
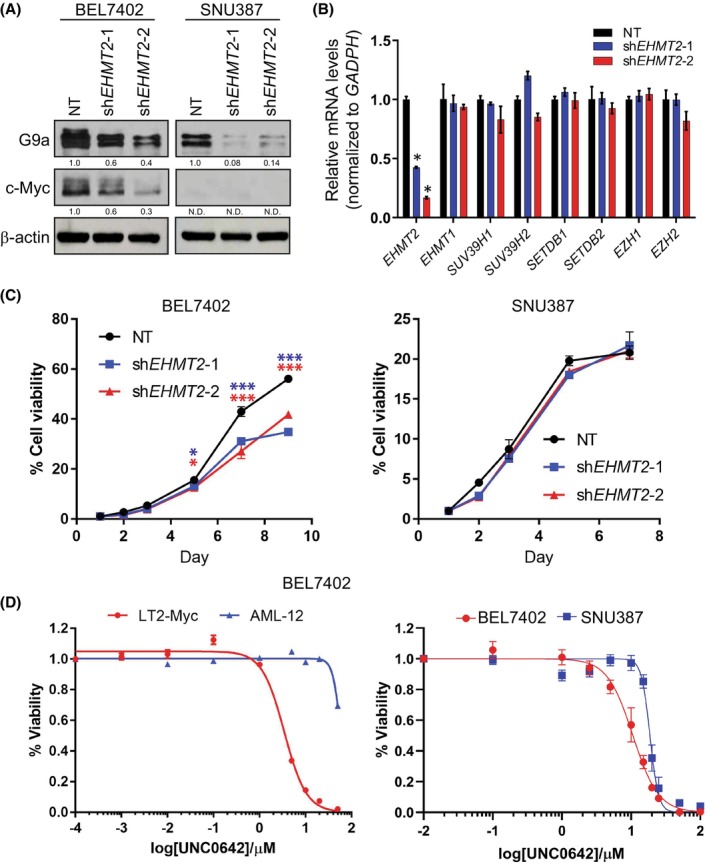
G9a inhibition reduces proliferation and Myc protein in hepatocellular carcinoma (HCC) cells. (A) Immunoblot analysis of G9a and c‐Myc levels in G9a‐depleted HCC cell lines (G9a^hi^ BEL7402 and G9a^lo^ SNU387) using two independent shRNAs, sh*EHMT2*‐1 and sh*EHMT2*‐2. β‐Actin was used as the loading control. Densitometry analyses for all proteins were normalised to β‐Actin and respective non‐targeting (NT) control cells. Analyses for c‐Myc in SNU387 were not determined (N.D.) due to the absence of a band. Densitometry analyses were represented as means below the immunoblots (*n* = 2). (B) Real‐time polymerase chain reaction of various H3K9me2‐related histone methyltransferases in G9a‐depleted BEL7402. *GAPDH* was used as the housekeeping internal control. Data represented as mean ± SD (*n* = 3). *P*‐values were determined by Student's *t*‐test. *P < 0.05. (C) Cell viability of G9a‐depleted BEL7402 (high endogenous G9a) (*left*) and SNU387 (low endogenous G9a) (*right*) using two independent shRNAs were assessed over a period of 9 and 7 days respectively. Data represented as mean ± SD (*n* = 3). *P*‐values were determined by Student's *t*‐test. *P < 0.05; ***P < 0.001. (D) Dose–response curves of murine and human liver cells treated with log concentrations of UNC0642 (*n* = 3).

To elucidate the relationships between G9a expression and proliferation in HCC, we first examined the growth of two HCC cells with different endogenous G9a expression by silencing G9a using two independent shRNAs (Fig. 3A). Importantly, we demonstrated that depletion of G9a resulted in a dose‐dependent decrease in the levels of the c‐Myc oncoprotein as well, further suggesting the positive correlation between both proteins as previously shown. The shRNAs targeting G9a specifically depleted G9a (*EHMT2*) but not GLP (*EHMT1*) and other histone methyltransferase mRNA expression (Fig. 3B). We showed that the proliferation rate of the high G9a HCC line, BEL7402 was significantly decreased over 9 days after G9a inhibition (Fig. 3C). However, G9a‐depletion had no significant effect on the low G9a and c‐Myc HCC line, SNU387. In addition, we also carried out a dose–response assay to determine the sensitivity of both human and murine liver cells towards pharmacological inhibition of G9a by UNC0642 and UNC0646 inhibitors. We showed that both the LT2‐Myc and BEL7402 (high endogenous G9a levels) tumour cells exhibited lower IC_50_ for both UNC0642 and UNC0646 compared to AML‐12 and SNU387 (low endogenous G9a levels; Fig. 3D, Fig. S3). Our results demonstrated that liver cells with high G9a expression are more susceptible to pharmacological inhibition of G9a.

### 3.5 G9a inhibition reduces migration and invasiveness of HCC cells

**Fig. 4:**
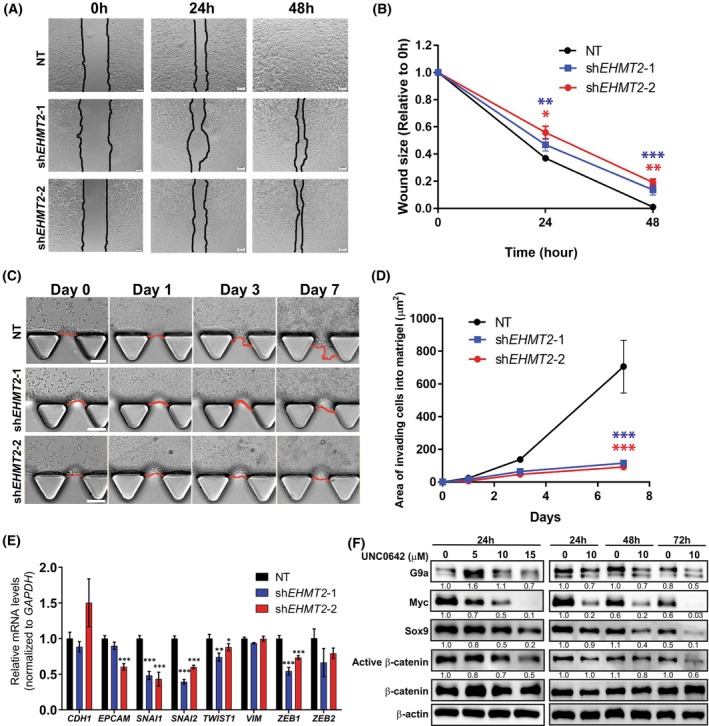
G9a inhibition reduces migration and invasiveness of hepatocellular carcinoma (HCC) cells. (A) Wound healing assay was used to determine the cell migration of BEL7402, the size of the wound areas was quantified in (B) between non‐targeting (NT), sh*EHMT2*‐1 and sh*EHMT2*‐2 cells. Scale bar denotes 50 μm. Data represented as mean ± SD (*n* = 3). *P*‐values were determined by Student's *t*‐test. *P < 0.05; **P < 0.01; ***P < 0.001. (C) Invasion ability of G9a‐depleted and NT BEL7402 cells was assessed using AIM Biotech Chip Invasion Assay (*n* = 3). (D) The reduced wound areas of cells that invaded into the Matrigel were monitored over 7 days and quantified by imagej analysis software (National Institutes of Health). Data represented as mean ± SD (*n* = 3). *P*‐values were determined by Student's *t*‐test. ***P < 0.001. (E) mRNA expression of epithelial‐mesenchymal transition (EMT)‐related genes in NT‐ and G9a‐depleted (sh*EHMT2*‐1 and sh*EHMT2*‐2) BEL7402 cells. Genes were normalised to housekeeping gene, *GAPDH*. Data represented as mean ± SD (*n* = 3). *P*‐values were determined by Student's *t*‐test. *P < 0.05; **P < 0.01; ***P < 0.001. (F) Immunoblot of Sox9/β‐catenin/Myc pathway post‐treatment with UNC0642 in a dose‐dependent manner, and at various time points (24‐, 48‐ and 72‐h). β‐Actin was used as the loading control. Densitometry analyses for all proteins were normalised to β‐Actin. Densitometry analyses were represented as means below the immunoblots (*n* = 3).

To elucidate the relationship between G9a and tumour cell invasiveness, we performed a wound healing assay on G9a‐NT and G9a‐depleted HCC cells. G9a‐depleted cells showed a significant reduction in the ability of the tumour cells to migrate after 48 h compared to the NT (Fig. 4A,B). In addition, we also carried out an AIM Chip invasion assay to determine the invasive potential of the HCC cells after G9a depletion. Importantly, we observed a significantly lower invasive potential of G9a‐depleted HCC cells compared to the G9a‐NT control (Fig. 4C,D). These data suggest the biological role of G9a in migration and invasion in HCC cells. To investigate if epithelial‐mesenchymal transition (EMT) markers were altered upon G9a depletion, we performed a qRT‐PCR of key EMT genes and observed that *SNAI1*, *SNAI2*, *ZEB1* and *TWIST1* were significantly reduced in G9a‐depleted HCC cells (Fig. 4E).

In addition, we explored the molecular pathways through which G9a inhibition mitigates the invasive capacity of HCC cells. Prior studies have demonstrated that the Sox9/β‐catenin/Myc axis contributes to the stemness properties of HCC cells and that the depletion of Sox9 was able to inhibit HCC metastasis both *in vitro* and *in vivo* [40]. Furthermore, inhibition of G9a in cholangiocarcinoma and colorectal cancer has been shown to reduce the levels of Sox9 [18, 41]. To investigate the consequences of G9a inhibition on the Sox9/β‐catenin/Myc axis in HCC, cells were treated with varying doses of G9a inhibitor, UNC0642, and at varying time points. Immunoblot analyses demonstrated that UNC0642 successfully suppressed the Sox9/β‐catenin/Myc pathway activation with a decrease in expression levels of all three proteins in a dose‐dependent and time‐dependent fashion (Fig. 4F). This suggests that G9a may play a role in promoting the invasive nature of HCC by activating the Sox9/β‐catenin/Myc axis.

### 3.6 Synthetic lethality between UNC0642 and Dinaciclib

Small molecule inhibitors of G9a histone methyltransferase have been shown to reduce HCC tumour volume *in vivo* and thus supported the use of G9a inhibitors as a novel therapeutic strategy for HCC [15]. To identify drug combinations effective against G9a^hi^/Myc^hi^ HCC tumours, we leveraged on quadratic phenotypic optimisation platform (QPOP), a computational algorithm which models the tumour response towards a panel of eight anti‐cancer drugs based on 91 combinations derived via OACD (Table S2). Cells were screened against the eight drugs and the IC_15_ and IC_30_ concentrations were established from the dose responses of the cells to the drugs (Fig. S4 and Table S3). Using the cell response to the 91 combinations, respective quadratic regression models were established for the different cell lines and HCC‐PDXOs to model their response to the eight drugs at three different doses (Table S4). QPOP performed in HCC cell lines demonstrated that dinaciclib and UNC0642 was an effective combination in G9a^hi^/Myc^hi^ lines BEL7402 and SNU398 as opposed to G9a^lo^/Myc^lo^ line SNU387 (Fig. S5A–D). An increase in efficacy was observed for BEL7402 and SNU398 as dosing levels of both drugs increased (Fig. S5B,C), but not for SNU387 (Fig. S5D).

**Fig. 5:**
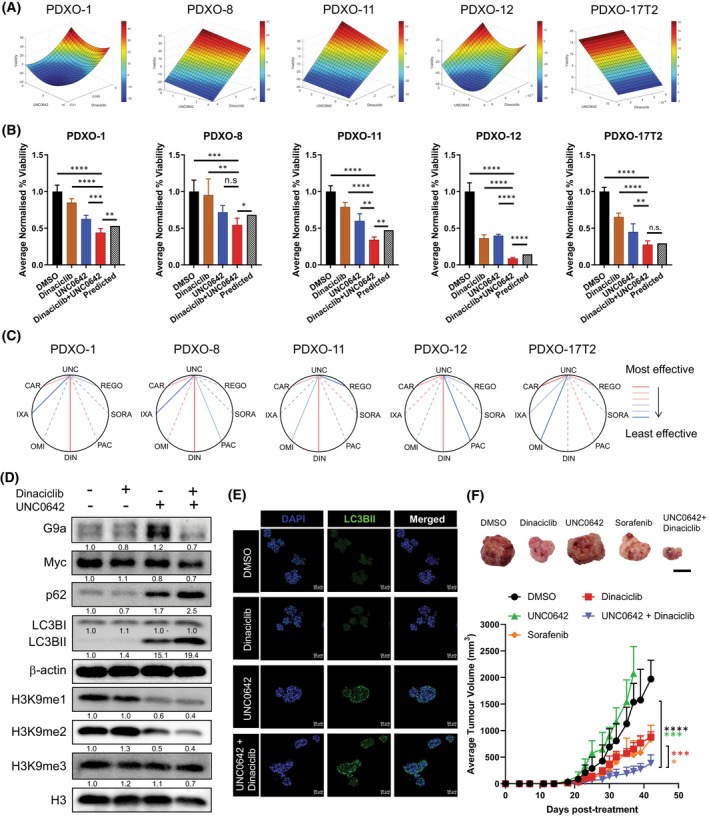
UNC0642 and dinaciclib promote autophagic cell death and inhibit hepatocellular carcinoma (HCC) tumorigenicity in patient‐derived avatars. (A) Parabolic surface response maps between UNC0642 and dinaciclib for five HCC‐patient‐derived xenograft organoid (PDXO) lines via quadratic phenotypic optimisation platform (QPOP). (B) Efficacy of UNC0642 and dinaciclib in reducing cell viability in five HCC‐PDXO lines over a period of 48 h. Cell viability of the organoids was normalised to the DMSO‐treated control group. Data represented as mean ± SD (*n* = 4). *P*‐values between all treatment groups were determined by Bonferroni‐corrected one‐way ANOVA; *P*‐values between the dinaciclib+UNC0642 treatment group and the predicted viability were determined by Student's *t*‐test. *P < 0.05; **P < 0.01; ***P < 0.001; ****P < 0.0001; n.s = no significance. (C) Polygonograms for the seven UNC0642‐based two‐drug combinations based on the QPOP analyses for five HCC‐PDXO lines. Effectiveness of each combination is represented by the lines in the following order in decreasing efficiency: Red solid, pink solid, pink dotted, light blue dotted, light blue solid, dark blue solid (*n* = 3). (D) Immunoblot analyses of autophagy markers, p62 and LC3B‐II were performed on PDXO‐11 organoids treated with UNC0642 and dinaciclib over 48 h. β‐Actin was used as the loading control. Densitometry analyses for all proteins and histone marks were normalised to β‐Actin and total histone 3 (H3) respectively. Densitometry analyses were represented as means below the immunoblots (*n* = 3). (E) Immunofluorescent staining of nuclear DAPI and LC3BII in PDXO‐11 following treatment with dinaciclib and UNC0642 (*n* = 3). Scale bar denotes 50 μm. (F) Representative images of PDXO‐11 subcutaneous tumours upon termination of drug treatment (*top*). Scale bar denotes 1 cm. Effect of sorafenib (*n* = 4), dinaciclib (*n* = 5) and UNC0642 (*n* = 5) in single and combination (*n* = 4) treatments on the growth of PDXO‐11 engrafted tumours in nude mice (*bottom*). (DMSO, *n* = 5) Data represented as mean ± SD. *P*‐values were determined by Student's *t*‐test. *P < 0.05; ***P < 0.001; ****P < 0.0001.

In a more clinically relevant *in vitro* model, QPOP analyses performed on HCC‐PDXOs exhibited high concordance with the cell lines. G9a^hi^/Myc^hi^ PDXO lines generated similar response surface maps, demonstrating the efficacy of dinaciclib and UNC0642 in targeting HCC (Fig. 5A, Fig. S5E). The efficacy of the combination was further determined using the bliss independence model in which the combination led to a greater reduction in organoid viability than expected (Fig. 5B). Efficacy of UNC0642 and dinaciclib, however, were limited in G9a^lo^/Myc^lo^ PDXO‐17T2, where the cells were unresponsive to increasing concentrations of dinaciclib (Fig. 5A,B, Fig. S5E). Comparisons of the top‐ranked two‐drug combinations across the PDXO lines demonstrated that dinaciclib with UNC0642 was amongst the top ranked combinations for G9a^hi^/Myc^hi^ lines, while regorafenib offered a more effective therapy option for PDXO‐17T2 (Table 1 and Table S5). Notably, amongst the seven drugs in the panel, dinaciclib was predicted to demonstrate the best efficacy with UNC0642 for G9a^hi^/Myc^hi^ PDXO lines, but not for G9a^lo^/Myc^lo^ PDXO‐17T2 (Fig. 5C). Furthermore, UNC0642 in combination with dinaciclib led to a reduction in protein levels of c‐Myc in G9a^hi^/Myc^hi^ PDXO‐11 as compared to their respective monotherapies (Fig. 5D). Interestingly, an increase in G9a levels was observed in PDXO‐11 when treated with UNC0642 alone, but reversed when UNC0642 was administered in combination with dinaciclib (Fig. 5D). We attribute this to a likely homeostatic response to a low dose of the G9a inhibitor as observed previously (Fig. 4F). The efficacy of dinaciclib and UNC0642 in targeting G9a^hi^/Myc^hi^ tumours was attributed to an increase in autophagic cell death. A significant increase in autophagy markers p62 and LC3B‐II was observed in PDXO‐11 treated with both dinaciclib and UNC0642 (Fig. 5D,E). Similarly, a corresponding upregulation of autophagy genes was observed (Fig. S5F). However, minimal upregulation of apoptosis was observed in the treated HCC‐PDXOs, suggesting that cell death induced by dinaciclib and UNC0642 was contingent on the activation of autophagy (Fig. S5G).

**Table 1:** Top‐ranked two‐drug combinations in G9a^Hi^/Myc^Hi^ PDXO‐1 and PDXO‐11, and G9a^Lo^/Myc^Lo^ PDXO‐17T2. Overall rankings are in parentheses.

Ranked	Dinaciclib	Carfilzomib	Ixazomib	Omipalisib	Pacritinib	Sorafenib	Regorafenib	UNC0642	Output	
Top‐ranked 2‐drug combinations (PDXO‐1)										
1 (2512)	0.01010	0	0	0	0.937	0	0	0	0.120	
2 (2521)	0.01010	0	0	0	0	0	0	6.313	0.121	Dinaciclib + UNC0642
3 (3310)	0.01010	0	0	0.162	0	0	0	0	0.199	
4 (3418)	0.00389	1.34586	0	0	0	0	0	0	0.209	
5 (3562)	0.01010	1.34586	0	0	0	0	0	0	0.224	
6 (3856)	0.01010	0	0	0	0	0	0	9.302	0.255	Dinaciclib + UNC0642
7 (3934)	0.01010	0.75723	0	0	0	0	0	0	0.262	
8 (3946)	0.01010	0	0	0	1.305	0	0	0	0.264	
9 (4114)	0.01010	0	0	0.033	0	0	0	0	0.288	
10 (4289)	0.01010	0	0	0	0	3.425	0	0	0.310	
(6528)	0	0	0	0	0	4.386	0	0	1.000	Sorafenib
(6556)	0	0	0	0	0	0	8.134	0	1.048	Regorafenib
Top‐ranked 2‐drug combinations (PDXO‐11)										
1 (3059)	0.00426	0	0	0	0.750	0	0	0	0.034	
2 (3441)	0.00426	0	0	0	0	4.751	0	0	0.072	
3 (4120)	0.00426	0	0	0	0.518	0	0	0	0.147	
4 (4183)	0.00426	0	0.01818	0	0	0	0	0	0.154	
5 (4258)	0.00426	0	0	0	0	5.568	0	0	0.162	
6 (4628)	0.00426	0	0	0	0	0	0	7.746	0.207	Dinaciclib + UNC0642
7 (4786)	0	0.00814	0	0	0.750	0	0	0	0.227	
8 (4801)	0.00426	0.00395	0	0	0	0	0	0	0.228	
9 (4812)	0.00426	0	0	0	0	0	0	6.812	0.230	Dinaciclib + UNC0642
10 (4824)	0.00426	0	0.02520	0	0	0	0	0	0.232	
(6511)	0	0	0	0	0	5.568	0	0	0.980	Sorafenib
(6561)	0	0	0	0	0	0	7.041	0	1.325	Regorafenib
Top‐ranked 2‐drug combinations (PDXO‐17T2)										
1 (2571)	0	0.21764	0	0	0	0	7.531	0	0.313	
2 (3532)	0	0.21764	0	0	0	0	5.230	0	0.408	
3 (3762)	0	0.21764	0	0	0.485	0	0	0	0.434	
4 (3887)	0	0.06749	0	0	0	0	7.531	0	0.447	
5 (4110)	0	0.21764	0	0	0	6.536	0	0	0.470	
6 (4155)	0	0.21764	0.08988	0	0	0	0	0	0.476	
7 (4210)	0	0.21764	0	0	0.364	0	0	0	0.482	
8 (4393)	0	0	0	0	0	6.536	7.531	0	0.502	
9 (4896)	0	0.06749	0	0	0	0	5.230	0	0.560	
10 (4957)	0	0.21764	0	0	0	0	0	9.638	0.566	
(6454)	0.00261	0	0	0	0	0	0	9.638	0.946	Dinaciclib + UNC0642
(6389)	0	0	0	0	0	6.536	0	0	0.898	Sorafenib
(5855)	0	0	0	0	0	0	7.531	0	0.721	Regorafenib

The efficacy of drug combination UNC0642 and dinaciclib was then validated in PDX models of HCC expressing high levels of G9a. Immunocompromised mice were grafted with HCC‐PDX cells subcutaneously, and subjected to treatment with UNC0642 and dinaciclib as monotherapies and in combination. A sorafenib‐treatment group was also included to compare the efficacy of the drug combination to the current standard of care for HCC patients. We observed that monotherapy treatment of UNC0642 alone was unable to reduce tumour growth in PDX models of HCC, demonstrating that inhibiting G9a alone was insufficient in mitigating tumour progression of HCC (Fig. 5F). Furthermore, the limited efficacy of UNC0642 *in vivo* can also be attributed to the poor solubility of the drug in water and hence poor bioavailability. Importantly, when we treated the tumour‐bearing mice with the synergistic drug combination (UNC0642 and dinaciclib), we showed that the optimal drug combination group decreased tumour volume as compared to the drugs administered singly or control, suggesting that dinaciclib can sensitise the tumours to UNC0642 and enhance its limited potency (Fig. 5F). The drug combination was also superior to the standard of care, sorafenib‐treated group, demonstrating a higher efficacy in mitigating tumour growth in HCC. Our data, therefore, show the enhanced tumour mitigating efficacy of the optimised drug combination of UNC0642 and dinaciclib in liver cancer.

## 4 Discussion

In this study, we identified G9a as an important modulator with a strong association with the oncogenic protein c‐Myc in maintaining the tumorigenicity in hepatocellular carcinoma. G9a has been shown to be upregulated in many solid cancers including head and neck, lung, ovarian, bladder and liver cancers. A comprehensive study by Wei et al. demonstrated the role of G9a in liver cancer progression via epigenetic silencing of tumour suppressor *RARRES3* [15]. Similarly, our work has shown that G9a is upregulated in liver cancer and targeting G9a is an epigenetic therapeutic option for the treatment of liver cancer. Our work here emphasises the role of G9a in mediating oncogenic progression through stabilisation of the c‐Myc protein. We showed that by inhibiting G9a in HCC either by pharmacological or genetic means, we were able to observe a concomitant decrease in the c‐Myc protein. Therefore, inhibition of G9a in HCC overexpressing c‐Myc oncoprotein might be an alternate viable strategy to target c‐Myc since efforts to target c‐Myc with small molecules have been challenging [10]. As the sample size of 10 HCC‐PDXs in our study is relatively small and may not be representative of the heterogenous nature of the disease, our study provides a strong rationale for future studies to further probe into the association between G9a and c‐Myc in a larger and more representative cohort of HCC.

Several lines of evidence have shown that G9a can be a biomarker or a prognostic marker for cancer development. Importantly, elevated G9a expression has been demonstrated to correlate with poorer prognosis and clinical outcomes. For instance, in breast, lung, colorectal and ovarian cancers, high G9a expression were associated with shorter overall survival in patients and the depletion of G9a suppressed several cancer hallmarks such as proliferation, invasiveness and tumour maintenance [13, 14, 18, 42]. Consistent with previous reports, we observed that high G9a protein expression can portend poor survival in HCC patients. Furthermore, to study the aggressive nature of tumours associated with high G9a expression reported previously, we showed that depleting G9a in HCC can also reduce the proliferative, invasive and migration abilities of cancer cells [13, 14, 20]. Importantly, we also showed that G9a and c‐Myc cooperate to predict for poorer prognostic outcome, suggesting that both G9a and c‐Myc may be used as prognostic markers of highly aggressive HCC.

Many anti‐cancer drugs are increasingly being tested in combination with other therapies, as it mitigates the risks of resistance developing in the tumours. Systemic therapies for HCC patients have also seen a turn towards combination therapies, with the recent approval of atezolizumab and bevacizumab as a standard of care for first‐line treatment of patients with unresectable advanced or metastatic HCC [4]. Recent developments vis‐à‐vis G9a‐targeted therapies have shifted towards combination therapies, where G9a and a secondary target are concurrently inhibited as a treatment strategy. This is in part also due to the poor solubility, limited bioavailability and modest efficacy of G9a inhibitors which limits their clinical efficacy as monotherapies, thus warranting a need for either development of better G9a inhibitors or combination therapies to enhance the limited efficacy of current G9a inhibitors [43, 44]. G9a have been shown to directly cooperate with DNA methyltransferase 1 (DNMT1) in coordinating epigenetic regulations necessary for replication during cell division [45]. Recent studies have demonstrated the efficacy of simultaneously attenuating G9a and DNMT1 activity in several cancer types with independent pharmacological inhibitors of both targets, and with a novel G9a/DNMT1 dual inhibitor, CM‐272 [46, 47, 48]. Combination therapies targeting G9a and EZH2 have also been gaining traction as G9a and EZH2 have been shown to regulate gene expression by cooperatively regulating H3K9 dimethylation and H3K27 trimethylation [49, 50, 51]. Subsequently, several groups have independently demonstrated the potential of G9a and EZH2 combination therapy as a viable treatment strategy in numerous cancer types [52, 53, 54]. Collectively, the evidence positions G9a‐based combination therapies as a promising strategy for development.

In this study, we identified the novel combination of UNC0642 and dinaciclib as an alternative strategy to target c‐Myc in HCC. Dinaciclib is amongst several CDK9 inhibitors which are currently in clinical trials, primarily in haematological malignancies [55]. Notably, the promise of dinaciclib has been demonstrated in patients with relapsed multiple myeloma, a cancer that is commonly driven by c‐Myc deregulation [56]. An overall partial response rate of 11% was observed in the patient cohort. The trial of several second‐generation CDK9 inhibitors such as BAY1143572 (NCT01938638), BAY1251152 (NCT02635672) and AZD4573 (NCT03263637) in patients with haematological malignancies which frequently exhibit a dependency on c‐Myc, such as multiple myeloma and diffuse large B‐cell lymphoma, further highlights the clinical potential of targeting CDK9 in Myc‐driven cancers [55, 57]. The pivotal role of CDK9 in Myc‐driven HCC have been established previously, where CDK9‐mediated expression of c‐Myc target genes is necessary for the maintenance of Myc‐driven HCC and the attenuation of CDK9 impaired Myc‐dependent oncogenesis [58]. This study therefore further reinforces the efficacy and value of CDK9 inhibitors in Myc‐dependent HCC, albeit concurrent with G9a inhibition. Importantly, we showed in a panel of clinically relevant patient‐derived avatars of HCC that the concurrent attenuation of the two vulnerabilities of c‐Myc, G9a and CDK9, was synergistic, reflecting the clinical relevance of this treatment strategy against Myc‐addicted HCC. Future studies can therefore be performed to further evaluate the potential of alternative CDK9 inhibitors in combination with G9a antagonists against Myc‐driven HCC.

As a key oncogenic driver of HCC, c‐Myc is an attractive target when designing treatment strategies for patients. Given the challenge in developing direct inhibitors of c‐Myc, vulnerabilities of c‐Myc have been identified across many cancer types, including HCC, providing alternative avenues to indirectly target c‐Myc in cancers [10]. Here, we demonstrate the cooperative relationship between epigenetic regulator G9a and c‐Myc in contributing to disease progression in HCC.

## 5 Conclusions

Our study suggests that G9a is an essential epigenetic dependency in Myc‐driven HCC, and provides a viable strategy against this oncogene addiction in HCC when combined with synthetic lethal targets of c‐Myc.

## Conflict of interest

EK‐HC and MBMAR are shareholders in KYAN Therapeutics. The other authors declare that they have no competing interest.

## Author contributions

DKHT, TBT and EK‐HC were responsible for project conceptualisation and manuscript preparations. DKHT, LH, CCMT, JJL, DR, IQCS and ZMT were responsible for performing experiments, data collection and analyses. DR and SJ were responsible for design of ChIP and PLA experiments. MBMAR was responsible for generating the code for QPOP analyses. LZ, AWCK, GKB, BKPG, JHK, YYD and PKHC were responsible for patient recruitment and performing the surgeries for the provision of patient material. TBT and EK‐HC were responsible for project supervision and administration. TBT, EK‐HC and PKHC were responsible for funding acquisition.

## Supporting information


**Fig. S1.** Correlation between G9a and Myc expression in hepatocellular carcinoma (HCC)‐patient‐derived xenografts (PDXs).
**Fig. S2.** Myc interacts with G9a in hepatocellular carcinoma (HCC).
**Fig. S3.** Inhibition of G9a in liver cell lines.
**Fig. S4.** Dose–response curves of hepatocellular carcinoma (HCC)‐patient‐derived xenograft organoids (PDXOs) and cell lines to a panel of eight anti‐cancer drugs.
**Fig. S5.** UNC0642 and dinaciclib promote autophagic cell death in hepatocellular carcinoma (HCC).
**Table S1.** List of primer sequences.
**Table S2.** Quadratic phenotypic optimisation platform (QPOP) combination design using orthogonal array composite design (OACD) consisting of 91 combinations.
**Table S3.** Concentrations of eight drugs used for quadratic phenotypic optimisation platform (QPOP) analyses in five hepatocellular carcinoma (HCC)‐patient‐derived xenograft organoid (PDXO) lines and three HCC cell lines.
**Table S4.** Parameter estimates and significance of quadratic phenotypic optimisation platform (QPOP) analyses on five hepatocellular carcinoma (HCC)‐patient‐derived xenograft organoid (PDXO) lines and three HCC cell lines.
**Table S5.** Top‐ranked two‐drug combinations in G9a^Hi^/Myc^Hi^ PDXO‐8 and PDXO‐12.

## Data Availability

Supplementary information is enclosed in supplementary figures and tables. The data that support the findings of this study are available from the corresponding author upon reasonable request.
