# Supplementary material for: Histone‐lysine N‐methyltransferase EHMT2 (G9a) inhibition mitigates tumorigenicity in Myc‐driven liver cancer
Source: Mol Oncol. 2023 Mar 29;17(11):2275–94. doi: 10.1002/1878-0261.13417 (PMC10620125; doi:10.1002/1878-0261.13417)
Supplement: Supplementary file 1 — Fig. S1. Correlation between G9a and Myc expression in hepatocellular carcinoma (HCC)‐patient‐derived xenografts (PDXs). Fig. S2. Myc interacts with G9a in hepatocellular carcinoma (HCC). Fig. S3. Inhibition of G9a in liver cell lines. Fig. S4. Dose–response curves of hepatocellular carcinoma (HCC)‐patient‐derived xenograft organoids (PDXOs) and cell lines to a panel of eight anti‐cancer drugs. Fig. S5. UNC0642 and dinaciclib promote autophagic cell death in hepatocellular carcinoma (HCC). Table S1. List of primer sequences. Table S2. Quadratic phenotypic optimisation platform (QPOP) combination design using orthogonal array composite design (OACD) consisting of 91 combinations. Table S3. Concentrations of eight drugs used for quadratic phenotypic optimisation platform (QPOP) analyses in five hepatocellular carcinoma (HCC)‐patient‐derived xenograft organoid (PDXO) lines and three HCC cell lines. Table S4. Parameter estimates and significance of quadratic phenotypic optimisation platform (QPOP) analyses on five hepatocellular carcinoma (HCC)‐patient‐derived xenograft organoid (PDXO) lines and three HCC cell lines. Table S5. Top‐ranked two‐drug combinations in G9aHi/MycHi PDXO‐8 and PDXO‐12. [file MOL2-17-2275-s001.docx]

Supplementary Materials for

**Histone-lysine N-methyltransferase *EHMT2* (G9a) inhibition as a therapeutic strategy against Myc-driven liver cancer**

Dexter Kai Hao Thng, Lissa Hooi, Clarissa Chin Min Toh, Jhin Jieh Lim, Deepa Rajagopalan, Imran Qamar Charles Syariff, Zher Min Tan, Masturah Bte Mohd Abdul Rashid, Lei Zhou, Alfred Wei Chieh Kow, Glenn Kunnath Bonney, Brian Kim Poh Goh, Juinn Huar Kam, Sudhakar Jha, Yock Young Dan, Pierce Kah Hoe Chow, Tan Boon Toh*, and Edward Kai-Hua Chow*

*Corresponding authors emails: [lsittb@nus.edu.sg](mailto:lsittb@nus.edu.sg) and [csikce@nus.edu.sg](mailto:csikce@nus.edu.sg)

**This file includes:**

Supplementary Figures 1 to 5

Supplementary Tables 1 to 5

**Supplementary Figures**

**
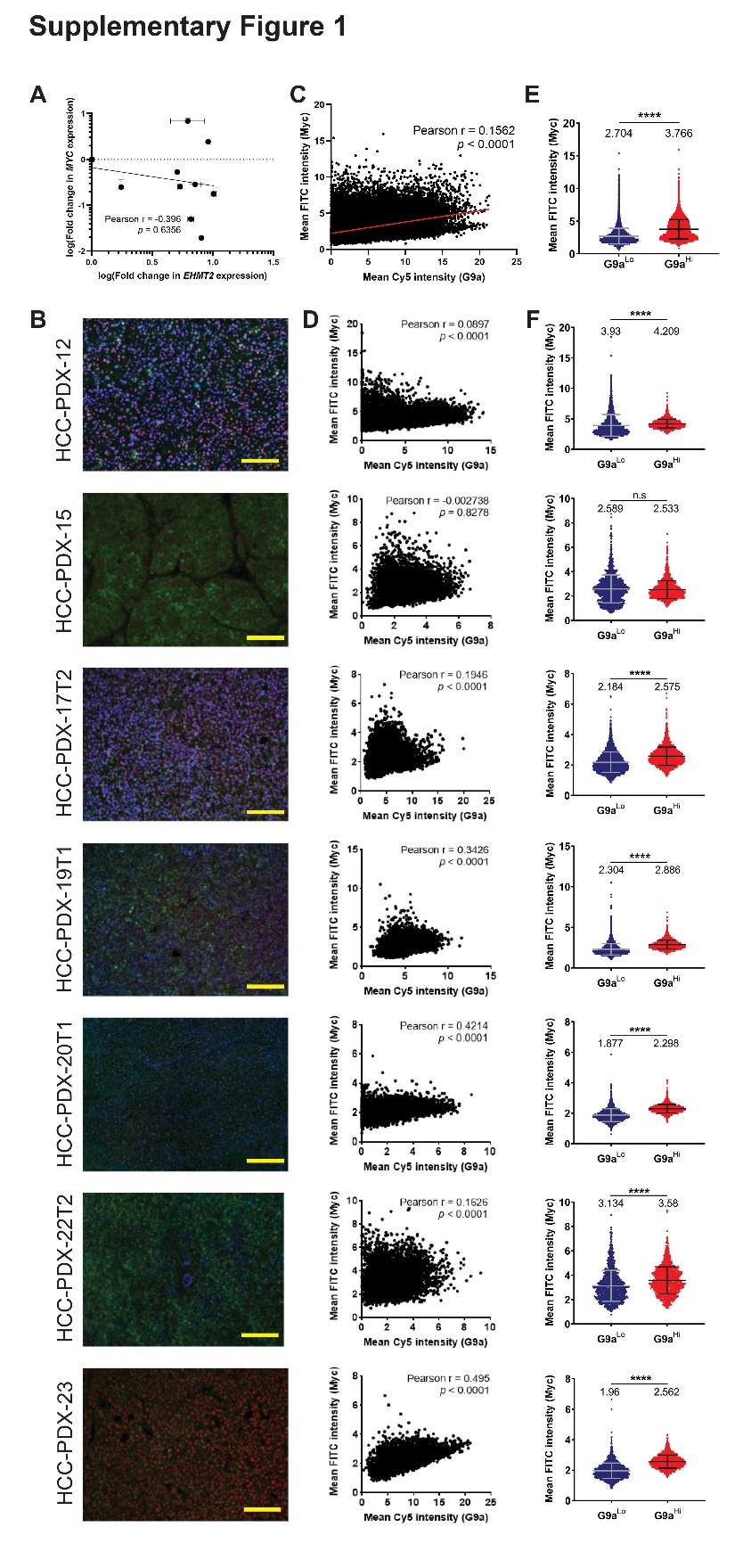
**

**Supplementary Figure 1. Correlation between G9a and Myc expression in hepatocellular carcinoma (HCC)-patient-derived xenografts (PDXs).**

**(A)** Relative transcript levels of *MYC* and *EHMT* in 10 HCC-PDX tissues. Genes were normalized to housekeeping gene, *GAPDH*.  **(B)** Multi-spectral imaging showing immunofluorescence co-staining of DAPI (blue), c-Myc (green) and G9a (red) in seven HCC patient-derived xenograft (PDX) tissues (n=5). **(C)** Scatter plots of G9a and c-Myc intensity collectively across 10 HCC patient-derived xenograft (PDX) tissues from **Figure 1F** and **Supplementary Figure 1B**. **(D)** Scatter plots of G9a and c-Myc intensity in the seven individual HCC PDX tissues showing positive correlation. **(E)** Staining intensity of c-Myc in G9a^hi^ and G9a^lo^ cells collectively across the 10 HCC PDX tissues and in **(F)** the seven individual HCC PDX tissues. Data represented as mean ± SD. p-values were determined by Student’s t-test. ****, *p* < 0.0001; n.s = no significance.

**
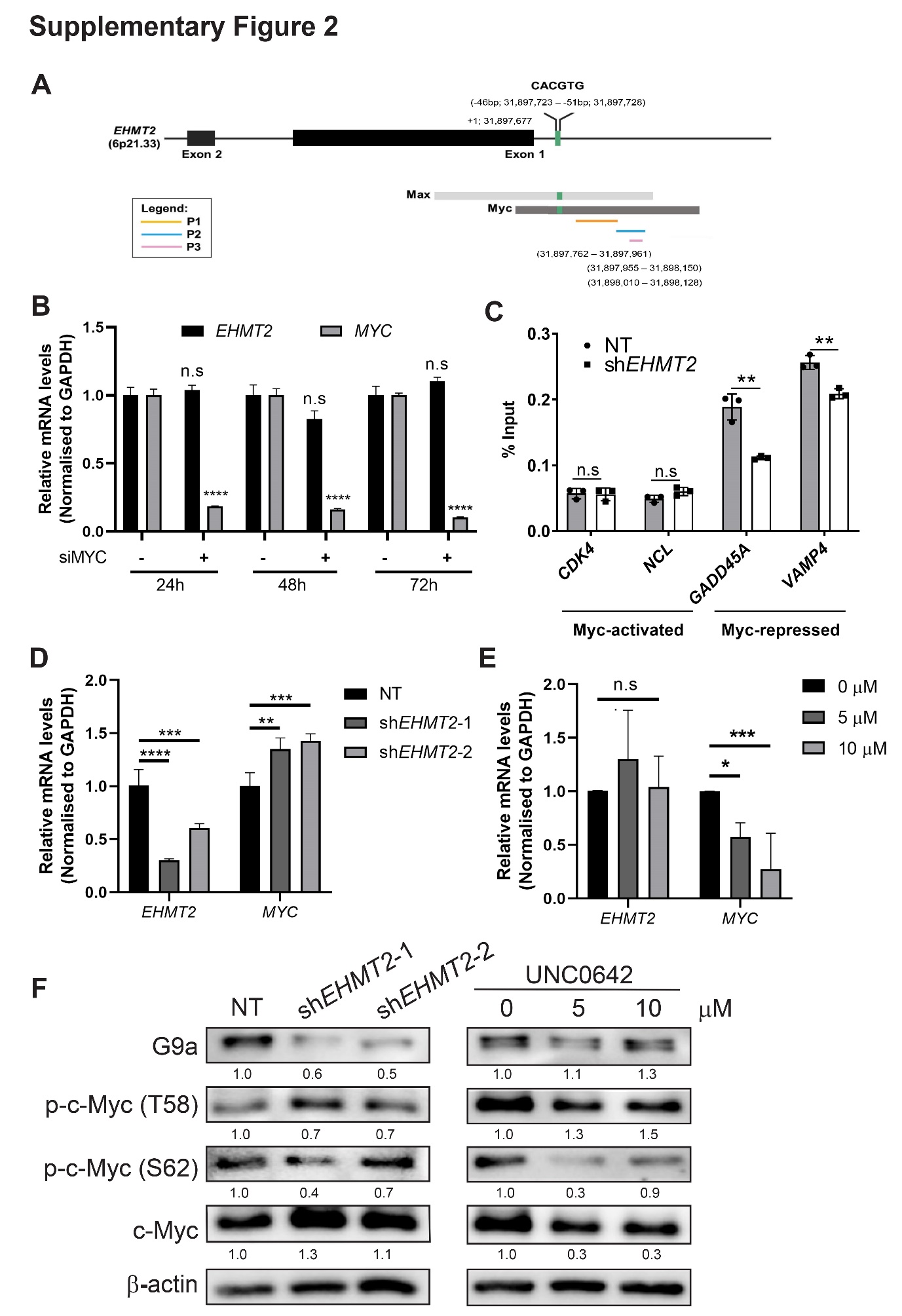
**

**Supplementary Figure 2. Myc interacts with G9a in hepatocellular carcinoma (HCC).**

**(A)** Schematic showing the relative region of *EHMT2* promoter amplified by real time-polymerase chain raction (RT-PCR) following chromatin immunoprecipitation (ChIP) assay with anti-c-Myc antibody. P1 to P3 span approximately 100-500 bp upstream of the transcriptional start site. **(B)** Transcript levels of *EHMT2* and *MYC* following transient c-Myc knockdown via small interfering RNA transfection. Genes were normalized to housekeeping gene, *GAPDH*. Data represented as mean ± SD (n=3). p-values were determined by Student’s t-test. ****, *p* < 0.0001; n.s = no significance. **(C)** G9a ChIP-RT-PCR in NT and shG9a BEL7402 at both Myc-activated and Myc-repressed genes. Data represented as mean ± SD of % input (n=3). p-values were determined by Student’s t-test. **, *p* < 0.01; n.s = no significance. **(D)** *EHMT2* and *MYC* mRNA levels in NT- and G9a-depleted (sh*EHMT2*-1 and sh*EHMT2*-2) BEL7402 cells, and in **(E)** UNC0642-treated BEL7402 cells at 0, 5 and 10µM. Genes were normalized to housekeeping gene, *GAPDH*. Data represented as mean ± SD (n=3). p-values were determined by Student’s t-test. *, *p* < 0.05; **, *p* < 0.01; ***, *p* < 0.001; ****, *p* < 0.0001; n.s = no significance. **(F)** Phosphorylation status of c-Myc (p-c-Myc) at threonine 58 and serine 62 in G9a-depleted and UNC0642-treated BEL7402 cells were determined via immunoblot analyses. β-actin was used as the loading control. Densitometry analyses for c-Myc and G9a were normalized to β-actin, while p-c-Myc levels were normalized to total c-Myc levels. Densitometry analyses were represented as means below the immunoblots (n=3).

**
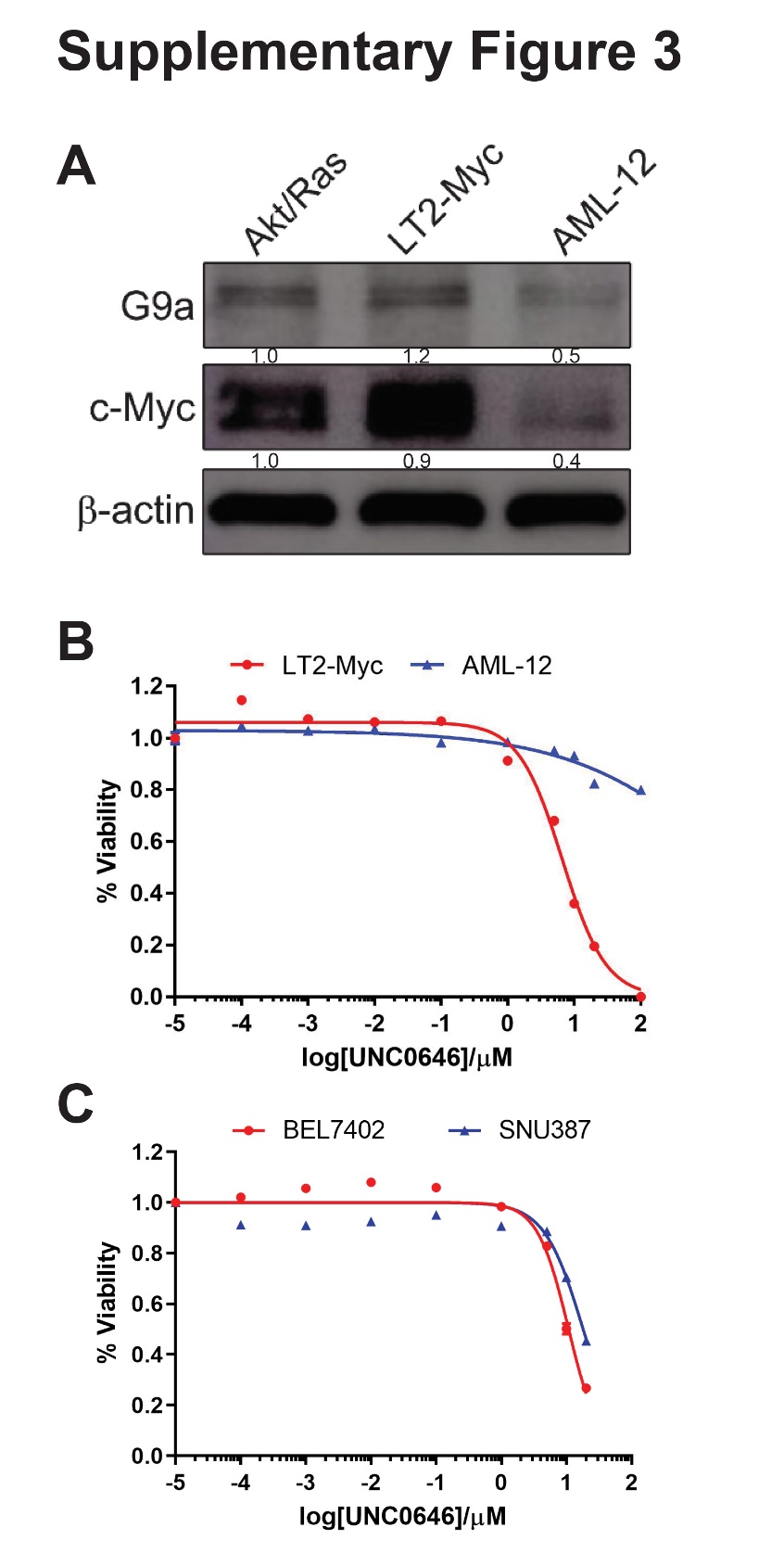
**

**Supplementary Figure 3. Inhibition of G9a in liver cell lines.**

**(A)** Immunoblot of G9a and c-Myc protein expression in Akt/Ras, LT2-Myc and AML-12 murine liver cell lines. β-actin was used as the loading control. Densitometry analyses for all proteins were normalized to β-actin. Densitometry analyses were represented as means below the immunoblots. **(B)** Dose-response curves of murine and **(C)** human liver cell lines treated with log concentrations of UNC0646.

**
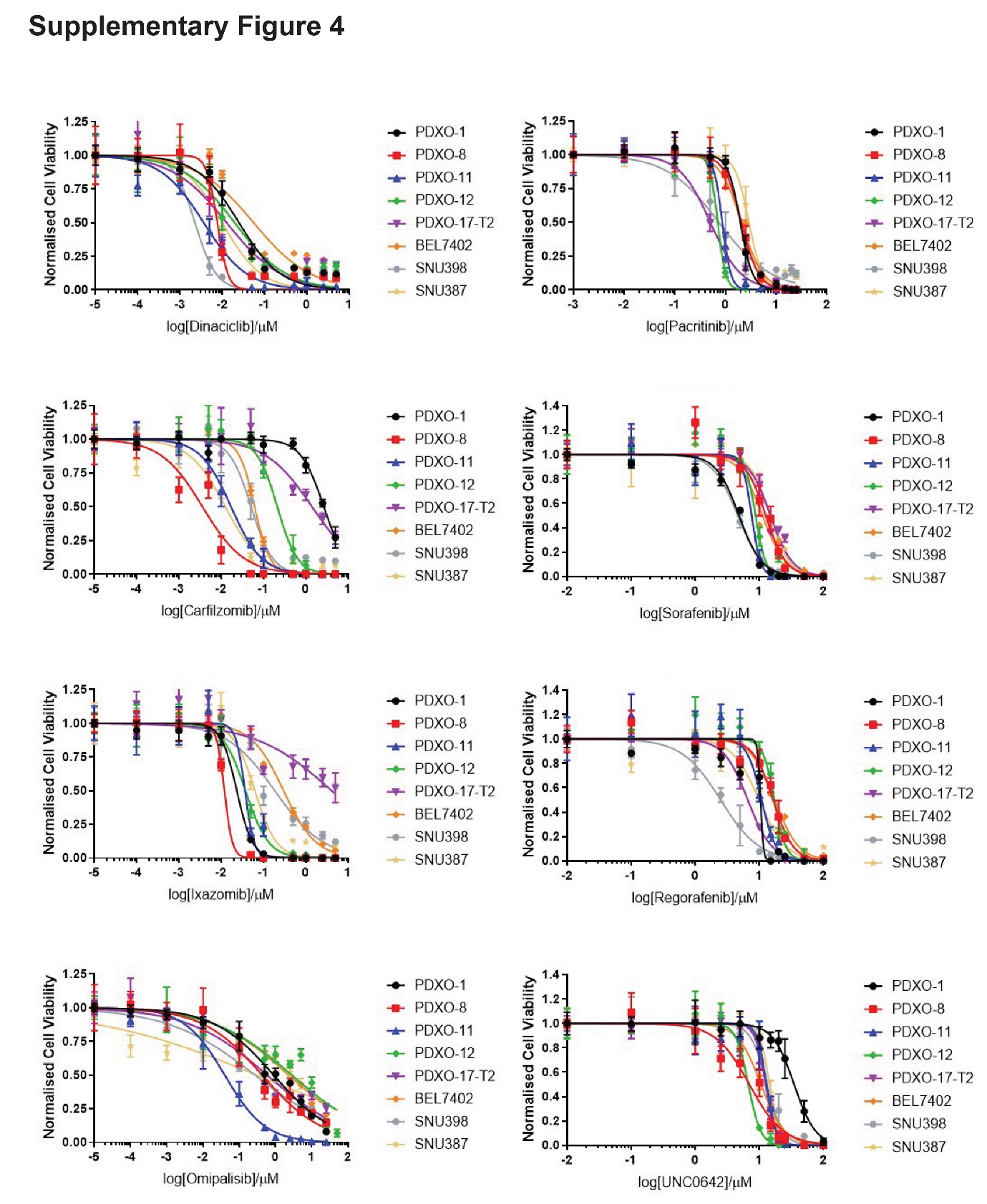
Supplementary Figure 4. Dose-response curves of hepatocellular carcinoma (HCC)-patient-drived xenograft organoids (PDXOs) and cell lines to a panel of eight anti-cancer drugs.**

Dose-response curves of five HCC-PDXO lines and three HCC cell lines to the panel of eight anti-cancer drugs included in quadratic phenotypic optimization platform (QPOP) experiments (dinaciclib, carfilzomib, ixazomib, omipalisib, pacritinib, sorafenib, regorafenib and UNC0642).

**
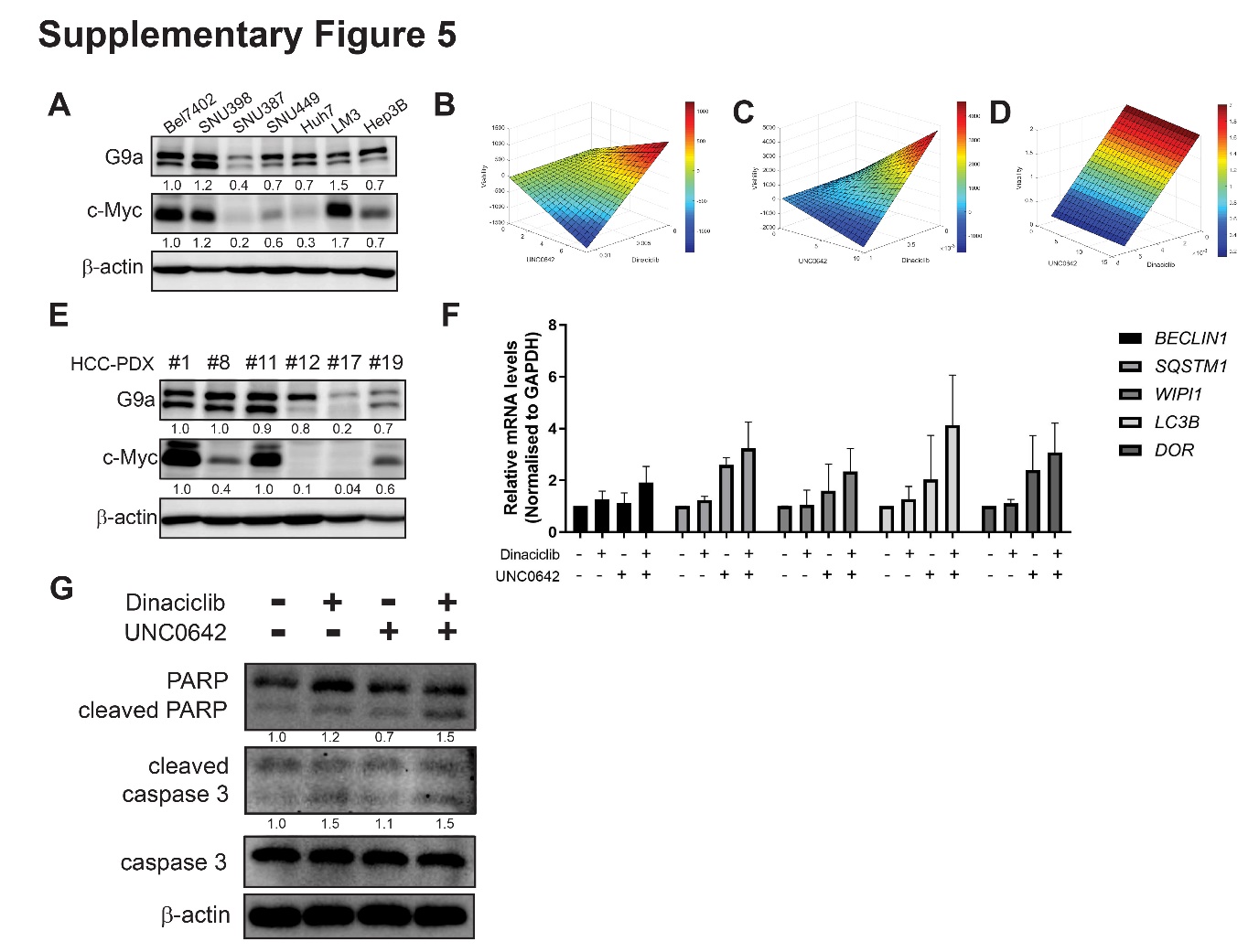
Supplementary Figure 5.** **UNC0642 and dinaciclib promotes autophagic cell death in hepatocellular carcinoma (HCC).**

**(A)** Immunoblot of G9a and Myc levels in a panel of seven human HCC cell lines. β-actin was used as the loading control. Densitometry analyses for all proteins were normalized to β-actin. Densitometry analyses were represented as means below the immunoblots (n=3). **(B)** Parabolic surface response maps of dinaciclib and UNC0642 illustrating the interaction of both drugs in G9a^Hi^/Myc^Hi^ cell lines BEL7402, **(C)** LM3, and **(D)** G9a^Lo^/Myc^Lo^ cell line, SNU387. **(E)** Immunoblot of G9a and Myc levels in a panel of six HCC-PDX lines. β-actin was used as the loading control. Densitometry analyses for all proteins were normalized to β-actin. Densitometry analyses were represented as means below the immunoblots (n=3). **(F)** Real time-polymerase chain reaction (RT-PCR) analysis of five autophagy associated genes in PDXO-11 treated with dinaciclib and UNC0642 singly and in combination. Data represented as mean ± SD (n=3). Genes were normalized to housekeeping gene, *GAPDH*. **(G)** Immunoblot of apoptosis markers, cleaved caspase 3 and cleaved PARP, in PDXO-11 treated with dinaciclib and UNC0642 singly and in combination. β-actin was used as the loading control. Densitometry analyses for all proteins were normalized to β-actin. Densitometry analyses were represented as means below the immunoblots (n=3).

**Supplementary Table 1. List of primer sequences.**

|  | Gene | Direction (5' → 3') | Primer sequence | Application |
| --- | --- | --- | --- | --- |
| Key targets | *EHMT1* | Forward | GCTGTG TGA AAA CCG AGC TG | qRT-PCR |
|  |  | Reverse | TCC GCT ATC CGA GTT AGT GTG |  |
|  | *EHMT2* | Forward | CTG TCA GAG GAG TTA GGT TCT GC |  |
|  |  | Reverse | CTT GCT GTC GGA GTC CAC G |  |
|  | *MYC* | Forward | AAA CAC AAA CTT GAA CAG CTA C |  |
|  |  | Reverse | ATT TGA GGC AGT TTA CAT TAT GG |  |
|  | *GAPDH* | Forward | AAG GTG AAG GTC GGA GTC AA |  |
|  |  | Reverse | AAT GAA GGG GTC ATT GAT GG |  |
| Epigenetic regulators | *EZH1* | Forward | GAG TTG GTC GAT GCC CTG AAT | qRT-PCR |
|  |  | Reverse | AGC ATG TCG CTT TCT CTT TCT T |  |
|  | *EZH2* | Forward | GGT GGG CGG CTT TCT TTA TCA |  |
|  |  | Reverse | GTA CAC GGG GAT AGA GAA TGT GG |  |
|  | *JHDM1D* | Forward | GTG GAG GTC CCT GAT ATA GCC |  |
|  |  | Reverse | CCA CCG AAG TCA ATG TGG AAA |  |
|  | *KDM1A* | Forward | TGA CCG GAT GAC TTC TCA AGA |  |
|  |  | Reverse | GTT GGA GAG TAG CCT CAA ATG TC |  |
|  | *KDM1B* | Forward | CTC TCC TGT GGG GAA CAT TTC |  |
|  |  | Reverse | GAC TAG GTT CGG TTT TGC CAT T |  |
|  | *KDM3A* | Forward | GTG GGA AAC AGC TCG AAT GGT |  |
|  |  | Reverse | GTG CTC ACG CTC GGA GAA A |  |
|  | *KDM3B* | Forward | TCT CCA GGG CAT TCG AGT CT |  |
|  |  | Reverse | TCT GAC AGG AAC CGA AGC TCT |  |
|  | *KDM4A* | Forward | ACT CCA GTG CTA GGA TAA TGA CC |  |
|  |  | Reverse | ACT CTT TTG GAG GAA CAA CCT TG |  |
|  | *KDM4B* | Forward | ACT TCA ACA AAT AG TGG CCT AC |  |
|  |  | Reverse | CGA TGT CAT CAT ACG TCT GCC |  |
|  | *KDM4C* | Forward | CGA GGT GGA AAG TCC TCT GAA |  |
|  |  | Reverse | GGG CTC CTT TAG ACT CCA TGT AT |  |
|  | *KDM4D* | Forward | ATG AAG GCG TCA ATA CAC CCT |  |
|  |  | Reverse | GGG GCA CCA CAT ACC AAG TT |  |
|  | *SETDB1* | Forward | TAA GAC TTG GCA CAA AGG CAC |  |
|  |  | Reverse | TCC CCG ACA GTA GAC TCT TTC |  |
|  | *SETDB2* | Forward | GGA GTC TAC GAA ACG TGG AGG |  |
|  |  | Reverse | CGA GCC AAC TGA ACA TAG GTA TT |  |
|  | *SUV39H1* | Forward | GCA GCG TGT GTT GCA AGT CTT |  |
|  |  | Reverse | CCA CGC CAT TTC ACC AGG TA |  |
|  | *SUV39H2* | Forward | CCA CCT GGT ACT CCC ATC TA |  |
|  |  | Reverse | TCT TTA CAC CCC AGC CAC GT |  |
| ChIP experiments | *NCL* | Forward | TTG CGA CGC GTA CGA GCT GG | ChIP-qPCR |
|  |  | Reverse | ACT CCG ACT AGG GCC GAT AC |  |
|  | *EHMT2 P1* | Forward | GGA GCC GCT ACA TGC TTT TT |  |
|  |  | Reverse | CAG AAG CTC CGC TTG TTA CCT |  |
|  | *EHMT2 P2* | Forward | GCT TCT GAA CTT CTC GGC TCT |  |
|  |  | Reverse | AAT AAA GGT GTC CTC CGC GT |  |
|  | *EHMT2 P3* | Forward | CTT CAT GTC CCC TGG AGC AG |  |
|  |  | Reverse | CTG CAG TGT CAC CAG TCA GT |  |
|  | *Chr6* | Forward | TGG CAT TGT CCT AAT ACT TCA GTG AT |  |
|  |  | Reverse | TTT CTG AAG TGC TGC TAC CTC TCA |  |
|  | *CDK4* | Forward | AGG CAT GTG TCA TGT GTG ATC TT |  |
|  |  | Reverse | CCG CTC CCA GTC TTC CTT G |  |
|  | *GADD45A* | Forward | AGA TGT GCT AGT TTC ATC ACC |  |
|  |  | Reverse | CCC TGC TAA AGG AAT TAG TCA C |  |
|  | *VAMP4* | Forward | CAG TGG TTG TTC CTC CCC T |  |
|  |  | Reverse | CCG AGC CCT ATT CAC CTA AA |  |
| EMT markers | *CDH1* | Forward | TTA CTG CCC CCA GAG GAT GA | qRT-PCR |
|  |  | Reverse | TGC AAC GTC GTT ACG AGT CA |  |
|  | *EPCAM* | Forward | GCT GGC CGT AAA CTG CTT TG |  |
|  |  | Reverse | ACA TTT GGC AGC CAG CTT TG |  |
|  | *SNAI1* | Forward | TCT TTC CTC GTC AGG AAG CC |  |
|  |  | Reverse | GAT CTC CGG AGG TGG GAT GG |  |
|  | *SNAI2* | Forward | CTC CTC ATC TTT GGG GCG AG |  |
|  |  | Reverse | CTT CAA TGG CAT GGG GGT CT |  |
|  | *TWIST1* | Forward | GTC CGC AGT CTT ACG AGG AG |  |
|  |  | Reverse | GCT TGA GGG TCT GAA TCT TGC T |  |
|  | *VIM* | Forward | CTG CCA ACC GGA ACA ATG AC |  |
|  |  | Reverse | CAT TTC ACG CAT CTG GCG TT |  |
|  | *ZEB1* | Forward | ATG CAG CTG ACT GTG AAG GT |  |
|  |  | Reverse | GAA AATGCA TCT GGT GTT CC |  |
|  | *ZEB2* | Forward | CCC TGG CAC AAC AAC GAG AT |  |
|  |  | Reverse | AAT TGC GGT CTG GAT CGT GG |  |
| Autophagy-associated genes | *BECLIN1* | Forward | GGT GTC TCT CGC AGA TTC ATC | qRT-PCR |
|  |  | Reverse | TCA GTC TTC GGC TGA GGT TCT |  |
|  | *DOR* | Forward | CTT CTG TCC TCA CTG TAC CTT G |  |
|  |  | Reverse | GAG AGG CGC TGG AAC ATG |  |
|  | *LC3B* | Forward | CCA GAT CCC TGC ACC ATG |  |
|  |  | Reverse | CTG CTT CTC ACC CTT GTA TCG |  |
|  | *SQSTM1* | Forward | GAC TAC GAC TTG TGT AGC GTC |  |
|  |  | Reverse | AGT GTC CGT GTT TCA CCT TCC |  |
|  | *WIPI1* | Forward | ACG GTG CCA GGT TAT TCT G |  |
|  |  | Reverse | CGT TTT GCC CTT CTG ATT TCC |  |

**Supplementary Table 2. Quadratic phenotypic optimization platform (QPOP) combination design using orthogonal array composite design (OACD) consisting of 91 combinations.**

| Combination No. | Dinaciclib | Carfilzomib | Ixazomib | Omipalisib | Pacritinib | Sorafenib | Regorafenib | UNC0642 |
| --- | --- | --- | --- | --- | --- | --- | --- | --- |
| 1 | -1 | -1 | -1 | -1 | -1 | -1 | -1 | 1 |
| 2 | -1 | -1 | -1 | -1 | -1 | 1 | -1 | -1 |
| 3 | -1 | -1 | -1 | -1 | 1 | -1 | 1 | 1 |
| 4 | -1 | -1 | -1 | -1 | 1 | 1 | 1 | -1 |
| 5 | -1 | -1 | -1 | 1 | -1 | -1 | 1 | 1 |
| 6 | -1 | -1 | -1 | 1 | -1 | 1 | 1 | -1 |
| 7 | -1 | -1 | -1 | 1 | 1 | -1 | -1 | 1 |
| 8 | -1 | -1 | -1 | 1 | 1 | 1 | -1 | -1 |
| 9 | -1 | -1 | 1 | -1 | -1 | -1 | 1 | -1 |
| 10 | -1 | -1 | 1 | -1 | -1 | 1 | 1 | 1 |
| 11 | -1 | -1 | 1 | -1 | 1 | -1 | -1 | -1 |
| 12 | -1 | -1 | 1 | -1 | 1 | 1 | -1 | 1 |
| 13 | -1 | -1 | 1 | 1 | -1 | -1 | -1 | -1 |
| 14 | -1 | -1 | 1 | 1 | -1 | 1 | -1 | 1 |
| 15 | -1 | -1 | 1 | 1 | 1 | -1 | 1 | -1 |
| 16 | -1 | -1 | 1 | 1 | 1 | 1 | 1 | 1 |
| 17 | -1 | 1 | -1 | -1 | -1 | -1 | 1 | -1 |
| 18 | -1 | 1 | -1 | -1 | -1 | 1 | 1 | 1 |
| 19 | -1 | 1 | -1 | -1 | 1 | -1 | -1 | -1 |
| 20 | -1 | 1 | -1 | -1 | 1 | 1 | -1 | 1 |
| 21 | -1 | 1 | -1 | 1 | -1 | -1 | -1 | -1 |
| 22 | -1 | 1 | -1 | 1 | -1 | 1 | -1 | 1 |
| 23 | -1 | 1 | -1 | 1 | 1 | -1 | 1 | -1 |
| 24 | -1 | 1 | -1 | 1 | 1 | 1 | 1 | 1 |
| 25 | -1 | 1 | 1 | -1 | -1 | -1 | -1 | 1 |
| 26 | -1 | 1 | 1 | -1 | -1 | 1 | -1 | -1 |
| 27 | -1 | 1 | 1 | -1 | 1 | -1 | 1 | 1 |
| 28 | -1 | 1 | 1 | -1 | 1 | 1 | 1 | -1 |
| 29 | -1 | 1 | 1 | 1 | -1 | -1 | 1 | 1 |
| 30 | -1 | 1 | 1 | 1 | -1 | 1 | 1 | -1 |
| 31 | -1 | 1 | 1 | 1 | 1 | -1 | -1 | 1 |
| 32 | -1 | 1 | 1 | 1 | 1 | 1 | -1 | -1 |
| 33 | 1 | -1 | -1 | -1 | -1 | -1 | 1 | -1 |
| 34 | 1 | -1 | -1 | -1 | -1 | 1 | 1 | 1 |
| 35 | 1 | -1 | -1 | -1 | 1 | -1 | -1 | -1 |
| 36 | 1 | -1 | -1 | -1 | 1 | 1 | -1 | 1 |
| 37 | 1 | -1 | -1 | 1 | -1 | -1 | -1 | -1 |
| 38 | 1 | -1 | -1 | 1 | -1 | 1 | -1 | 1 |
| 39 | 1 | -1 | -1 | 1 | 1 | -1 | 1 | -1 |
| 40 | 1 | -1 | -1 | 1 | 1 | 1 | 1 | 1 |
| 41 | 1 | -1 | 1 | -1 | -1 | -1 | -1 | 1 |
| 42 | 1 | -1 | 1 | -1 | -1 | 1 | -1 | -1 |
| 43 | 1 | -1 | 1 | -1 | 1 | -1 | 1 | 1 |
| 44 | 1 | -1 | 1 | -1 | 1 | 1 | 1 | -1 |
| 45 | 1 | -1 | 1 | 1 | -1 | -1 | 1 | 1 |
| 46 | 1 | -1 | 1 | 1 | -1 | 1 | 1 | -1 |
| 47 | 1 | -1 | 1 | 1 | 1 | -1 | -1 | 1 |
| 48 | 1 | -1 | 1 | 1 | 1 | 1 | -1 | -1 |
| 49 | 1 | 1 | -1 | -1 | -1 | -1 | -1 | 1 |
| 50 | 1 | 1 | -1 | -1 | -1 | 1 | -1 | -1 |
| 51 | 1 | 1 | -1 | -1 | 1 | -1 | 1 | 1 |
| 52 | 1 | 1 | -1 | -1 | 1 | 1 | 1 | -1 |
| 53 | 1 | 1 | -1 | 1 | -1 | -1 | 1 | 1 |
| 54 | 1 | 1 | -1 | 1 | -1 | 1 | 1 | -1 |
| 55 | 1 | 1 | -1 | 1 | 1 | -1 | -1 | 1 |
| 56 | 1 | 1 | -1 | 1 | 1 | 1 | -1 | -1 |
| 57 | 1 | 1 | 1 | -1 | -1 | -1 | 1 | -1 |
| 58 | 1 | 1 | 1 | -1 | -1 | 1 | 1 | 1 |
| 59 | 1 | 1 | 1 | -1 | 1 | -1 | -1 | -1 |
| 60 | 1 | 1 | 1 | -1 | 1 | 1 | -1 | 1 |
| 61 | 1 | 1 | 1 | 1 | -1 | -1 | -1 | -1 |
| 62 | 1 | 1 | 1 | 1 | -1 | 1 | -1 | 1 |
| 63 | 1 | 1 | 1 | 1 | 1 | -1 | 1 | -1 |
| 64 | 1 | 1 | 1 | 1 | 1 | 1 | 1 | 1 |
| 65 | -1 | -1 | -1 | -1 | -1 | -1 | -1 | -1 |
| 66 | -1 | -1 | 0 | 0 | -1 | 1 | 0 | 1 |
| 67 | -1 | -1 | 1 | 1 | -1 | 0 | 1 | 0 |
| 68 | -1 | 0 | -1 | 0 | 1 | 0 | -1 | 0 |
| 69 | -1 | 0 | 0 | 1 | 1 | -1 | 0 | -1 |
| 70 | -1 | 0 | 1 | -1 | 1 | 1 | 1 | 1 |
| 71 | -1 | 1 | -1 | 1 | 0 | 1 | -1 | 1 |
| 72 | -1 | 1 | 0 | -1 | 0 | 0 | 0 | 0 |
| 73 | -1 | 1 | 1 | 0 | 0 | -1 | 1 | -1 |
| 74 | 0 | -1 | -1 | 0 | 0 | 0 | 0 | -1 |
| 75 | 0 | -1 | 0 | 1 | 0 | -1 | 1 | 1 |
| 76 | 0 | -1 | 1 | -1 | 0 | 1 | -1 | 0 |
| 77 | 0 | 0 | -1 | 1 | -1 | 1 | 0 | 0 |
| 78 | 0 | 0 | 0 | -1 | -1 | 0 | 1 | -1 |
| 79 | 0 | 0 | 1 | 0 | -1 | -1 | -1 | 1 |
| 80 | 0 | 1 | -1 | -1 | 1 | -1 | 0 | 1 |
| 81 | 0 | 1 | 0 | 0 | 1 | 1 | 1 | 0 |
| 82 | 0 | 1 | 1 | 1 | 1 | 0 | -1 | -1 |
| 83 | 1 | -1 | -1 | 1 | 1 | 1 | 1 | -1 |
| 84 | 1 | -1 | 0 | -1 | 1 | 0 | -1 | 1 |
| 85 | 1 | -1 | 1 | 0 | 1 | -1 | 0 | 0 |
| 86 | 1 | 0 | -1 | -1 | 0 | -1 | 1 | 0 |
| 87 | 1 | 0 | 0 | 0 | 0 | 1 | -1 | -1 |
| 88 | 1 | 0 | 1 | 1 | 0 | 0 | 0 | 1 |
| 89 | 1 | 1 | -1 | 0 | -1 | 0 | 1 | 1 |
| 90 | 1 | 1 | 0 | 1 | -1 | -1 | -1 | 0 |
| 91 | 1 | 1 | 1 | -1 | -1 | 1 | 0 | -1 |
|  |  |  |  |  |  |  |  |  |

**Supplementary Table 3. Concentrations of eight drugs used for quadratic phenotypic optimization platform (QPOP) analyses in five hepatocellular carcinoma (HCC)-patient-derived xenograft organoid (PDXO) lines and three HCC cell lines.** IC_15_ and IC_30_ values are intrapolated from dose-response curves of HCC-PDXO lines and cell lines to log concentrations of the eight drugs in Supplementary Figure 5. Data are averages of three independent experiments.

|  |  | Dinaciclib | Carfilzomib | Ixazomib | Omipalisib | Pacritinib | Sorafenib | Regorafenib | UNC0642 |
| --- | --- | --- | --- | --- | --- | --- | --- | --- | --- |
| PDXO-1 | IC_50_ (µM) | 0.02514 | 2.33600 | 0.02358 | 0.750 | 1.801 | 5.563 | 9.450 | 13.470 |
|  | IC_15_ (µM) | 0.00389 | 0.75723 | 0.01195 | 0.033 | 0.937 | 3.425 | 7.113 | 6.313 |
|  | IC_30_ (µM) | 0.01010 | 1.34586 | 0.01691 | 0.162 | 1.305 | 4.386 | 8.134 | 9.302 |
| PDXO-8 | IC_50_ (µM) | 0.00705 | 0.00301 | 0.01539 | 0.301 | 2.395 | 8.800 | 17.235 | 6.695 |
|  | IC_15_ (µM) | 0.00269 | 0.00054 | 0.00982 | 0.010 | 1.352 | 3.821 | 9.809 | 2.462 |
|  | IC_30_ (µM) | 0.00413 | 0.00130 | 0.01235 | 0.056 | 1.809 | 5.842 | 13.087 | 4.106 |
| PDXO-11 | IC_50_ (µM) | 0.00634 | 0.01622 | 0.03491 | 0.025 | 1.108 | 6.490 | 9.208 | 8.787 |
|  | IC_15_ (µM) | 0.00289 | 0.00395 | 0.01818 | 0.003 | 0.518 | 4.751 | 5.318 | 6.812 |
|  | IC_30_ (µM) | 0.00426 | 0.00814 | 0.02520 | 0.009 | 0.750 | 5.568 | 7.041 | 7.746 |
| PDXO-12 | IC_50_ (µM) | 0.01586 | 0.16320 | 0.03449 | 4.166 | 0.991 | 8.371 | 14.950 | 7.093 |
|  | IC_15_ (µM) | 0.00262 | 0.05838 | 0.01296 | 0.285 | 0.549 | 4.619 | 8.273 | 4.629 |
|  | IC_30_ (µM) | 0.00625 | 0.09876 | 0.02132 | 1.206 | 0.737 | 6.232 | 11.155 | 5.758 |
| PDXO-17T2 | IC_50_ (µM) | 0.01299 | 0.95100 | 1.29210 | 0.514 | 0.596 | 11.262 | 9.976 | 14.655 |
|  | IC_15_ (µM) | 0.00164 | 0.06749 | 0.01362 | 0.001 | 0.364 | 4.645 | 5.230 | 8.529 |
|  | IC_30_ (µM) | 0.00261 | 0.21764 | 0.08988 | 0.013 | 0.485 | 6.536 | 7.531 | 9.638 |
| BEL7402 | IC_50_ (µM) | 0.04077 | 0.06404 | 0.18942 | 1.346 | 2.164 | 9.316 | 15.363 | 10.750 |
|  | IC_15_ (µM) | 0.00337 | 0.02734 | 0.03950 | 0.013 | 0.961 | 4.417 | 7.856 | 5.882 |
|  | IC_30_ (µM) | 0.01116 | 0.04220 | 0.09408 | 0.138 | 1.455 | 6.461 | 10.990 | 7.984 |
| SNU398 | IC_50_ (µM) | 0.00256 | 0.05650 | 0.26983 | 0.355 | 0.733 | 4.956 | 2.932 | 13.937 |
|  | IC_15_ (µM) | 0.00046 | 0.01092 | 0.01909 | 0.003 | 0.113 | 2.329 | 1.201 | 6.666 |
|  | IC_30_ (µM) | 0.00098 | 0.02479 | 0.07296 | 0.034 | 0.290 | 3.425 | 1.886 | 9.677 |
| SNU387 | IC_50_ (µM) | 0.01304 | 0.02190 | 0.11041 | 0.346 | 2.903 | 15.727 | 11.618 | 14.339 |
|  | IC_15_ (µM) | 0.00220 | 0.00734 | 0.02711 | 0.000 | 1.515 | 9.309 | 4.505 | 9.234 |
|  | IC_30_ (µM) | 0.00544 | 0.01280 | 0.05508 | 0.011 | 2.432 | 12.543 | 6.576 | 11.094 |

**Supplementary Table 4. Parameter estimates and significance of quadratic phenotypic optimization platform (QPOP) analyses on five hepatocellular carcinoma (HCC)-patient-derived xenograft organoid (PDXO) lines and three HCC cell lines.** Statistical analyses were performed using sum of squares F-test (*, p < 0.05; **, p < 0.01; ***, p < 0.001).

|  | PDXO-1 | | PDXO-8 | | PDXO-11 | | PDXO-12 | | PDXO-17T2 | | BEL7402 | | SNU398 | | SNU387 | |
| --- | --- | --- | --- | --- | --- | --- | --- | --- | --- | --- | --- | --- | --- | --- | --- | --- |
|  | Estimate | Significance | Estimate | Significance | Estimate | Significance | Estimate | Significance | Estimate | Significance | Estimate | Significance | Estimate | Significance | Estimate | Significance |
| Intercept | 1.000 | *** | 0.934 | *** | 1.218 | *** | 1.442 | *** | 1.117 | *** | 0.960 | *** | 0.938 | *** | 0.709 | *** |
| Dinaciclib | -146.233 | *** | -164.101 | *** | -191.925 | *** | -95.529 | *** | -42.500 | *** | -48.218 | *** | -141.591 |  | -27.867 | *** |
| Carfilzomib | -0.370 | *** | -281.903 | *** | -145.214 | *** | -4.223 | *** | -5.429 | *** | 1.443 |  | -1.079 |  | - | - |
| Ixazomib | 3.480 |  | 5.057 |  | -31.327 | * | -23.867 | *** | -0.716 | * | -5.193 | *** | -1.518 |  | -5.158 | * |
| Omipalisib | -0.687 | * | -19.529 | * | 6.843 |  | -0.282 | *** | 54.631 |  | -2.312 | *** | 1.529 |  | -12.429 | *** |
| Pacritinib | -0.629 |  | 0.000 |  | -0.488 | *** | -1.077 |  | -0.397 | *** | -0.622 | *** | -2.162 | ** | -0.183 | *** |
| Sorafenib | - | - | -0.011 |  | -0.222 | * | -0.176 | * | 0.014 |  | -0.096 | * | -0.118 | *** | -0.017 | *** |
| Regorafenib | 0.006 |  | -0.063 | * | 0.015 |  | -0.092 | * | -0.053 | *** | -0.129 | *** | -0.215 | *** | -0.020 | *** |
| UNC0642 | -0.081 |  | -0.026 | ** | -0.045 | *** | -0.232 | ** | -0.006 | *** | -0.010 | * | -0.020 | *** | -0.027 | *** |
| Dinaciclib:Carfilzomib | 30.295 | *** | 74340.878 | *** | 16091.545 | *** | 315.368 | * | 145.317 | * | -278.629 | * | -6717.873 | ** | - | - |
| Dinaciclib:Ixazomib | - | - | - | - | - | - | - | - | - | - | 355.637 | *** | - | - | - | - |
| Dinaciclib:Omipalisib | - | - | 619.739 |  | - | - | 33.372 | ** | - | - | 89.091 | * | - | - | 623.928 |  |
| Dinaciclib:Pacritinib | - | - | - | - | - | - | - | - | - | - | - | - | 376.194 |  | 5.622 | ** |
| Dinaciclib:Sorafenib | - | - | - | - | - | - | - | - | - | - | - | - | 36.199 | * | - | - |
| Dinaciclib:Regorafenib | - | - | - | - | - | - | - | - | - | - | 0.839 |  | - | - | - | - |
| Dinaciclib:UNC0642 | - | - | - | - | 4.646 |  | - | - | - | - | - | - | -10.775 |  | 1.098 | ** |
| Carfilzomib:Ixazomib | - | - | - | - | - | - | - | - | -4.421 | * | - | - | -98.361 | ** | - | - |
| Carfilzomib:Pacritinib | - | - | -100.245 | ** | - | - | - | - | - | - | - | - | - | - | - | - |
| Carfilzomib:Sorafenib | - | - | - | - | - | - | - | - | 0.044 |  | - | - | 1.541 | * | - | - |
| Carfilzomib:Regorafenib | - | - | 9.525 | * | - | - | - | - | 0.050 | * | - | - | 3.424 | ** | - | - |
| Ixazomib:Omipalisib | - | - | - | - | - | - | 7.529 | * | - | - | 21.108 | *** | -45.374 | * | - | - |
| Ixazomib:Pacritinib | - | - | - | - | 7.856 |  | - | - | - | - | 0.759 |  | - | - | - | - |
| Ixazomib:Sorafenib | - | - | - | - | 1.535 | ** | 1.770 | * | - | - | - | - | 0.527 | * | - | - |
| Ixazomib:Regorafenib | -1.498 | * | -1.019 | * | -0.748 |  | - | - | 0.127 | * | - | - | 1.179 | ** | - | - |
| Ixazomib:UNC0642 | - | - | - | - | - | - | 1.640 | * | - | - | - | - | 0.141 |  | - | - |
| Omipalisib:Pacritinib | - | - | - | - | - | - | - | - | - | - | - | - | - | - | 3.365 | *** |
| Omipalisib:Regorafenib | - | - | - | - | -2.509 |  | - | - | - | - | - | - | - | - | - | - |
| Omipalisib:UNC0642 | - | - | - | - | - | - | - | - | - | - | - | - | 0.324 |  | 0.344 | * |
| Pacritinib:Sorafenib | - | - | - | - | 0.038 | * | - | - | 0.020 |  | - | - | - | - | 0.004 | *** |
| Pacritinib:Regorafenib | - | - | - | - | - | - | - | - | 0.021 | * | - | - | - | - | 0.005 | ** |
| Pacritinib:UNC0642 | - | - | - | - | 0.036 | ** | - | - | - | - | - | - | - | - | 0.005 | *** |
| Sorafenib:Regorafenib | - | - | - | - |  |  | - | - | - | - | - | - | - | - | -0.001 |  |
| Sorafenib:UNC0642 | - | - | - | - |  |  | 0.006 | * | - | - | 0.002 |  | - | - | 0.001 | ** |
| Regorafenib:UNC0642 | - | - | - | - |  |  | - | - | - | - | - | - | - | - | 0.001 | * |
| Dinaciclib^2 | 7718.941 | * | - | - |  |  | - | - | - | - | - | - | 379316.687 |  | - | - |
| Carfilzomib^2 | - | - | - | - | 8413.712 | * | - | - | 14.583 | *** | - | - | - | - | - | - |
| Ixazomib^2 | - | - | - | - | 979.344 |  | - | - | - | - | - | - | - | - | 91.587 | * |
| Omipalisib^2 | - | - | 303.102 | * | - | - | - | - | -4259.690 |  | - | - | - | - | - | - |
| Pacritinib^2 | 0.455 |  | - | - | - | - | 1.470 |  | - | - | 0.398 | *** | 6.416 | ** | - | - |
| Sorafenib^2 | - | - | - | - | 0.032 |  | 0.019 |  | -0.007 | * | 0.012 | * | - | - | - | - |
| Regorafenib^2 | - | - | 0.004 | * | - | - | 0.008 | * | - | - | 0.010 | *** | - | - | - | - |
| UNC0642^2 | 0.008 |  | - | - | - | - | 0.029 |  | - | - | - | - | - | - | - | - |
| R2 (QPOP) | 0.834 | | 0.869 | | 0.918 | | 0.881 | | 0.971 | | 0.895 | | 0.910 | | 0.912 | |

**Supplementary Table 5. Top-ranked two-drugs combinations in G9a^Hi^/Myc^Hi^ PDXO-8 and PDXO-12.** Overall rankings are in parentheses.

| Top-ranked 2-drugs combinations (PDXO-8) | | | | | | | | | | |
| --- | --- | --- | --- | --- | --- | --- | --- | --- | --- | --- |
| Ranked | Dinaciclib | Carfilzomib | Ixazomib | Omipalisib | Pacritinib | Sorafenib | Regorafenib | UNC0642 | Output |  |
| 1 (2143) | 0.00413 | 0 | 0 | 0 | 0 | 0 | 9.809 | 0 | 0.050 |  |
| 2 (2912) | 0.00413 | 0 | 0 | 0.010 | 0 | 0 | 0 | 0 | 0.119 |  |
| 3 (3245) | 0.00413 | 0 | 0 | 0 | 0 | 0 | 0 | 4.106 | 0.150 | Dinaciclib + UNC0642 |
| 4 (3391) | 0.00413 | 0 | 0 | 0 | 0 | 0 | 13.087 | 0 | 0.164 |  |
| 5 (3641) | 0.00413 | 0 | 0 | 0 | 0 | 5.842 | 0 | 0 | 0.188 |  |
| 6 (3684) | 0.00413 | 0 | 0 | 0 | 0 | 0 | 0 | 2.462 | 0.192 | Dinaciclib + UNC0642 |
| 7 (3872) | 0.00413 | 0 | 0 | 0 | 0 | 3.821 | 0 | 0 | 0.211 |  |
| 8 (4257) | 0.00413 | 0 | 0 | 0 | 1.809 | 0 | 0 | 0 | 0.255 |  |
| 9 (4259) | 0.00413 | 0 | 0 | 0 | 1.352 | 0 | 0 | 0 | 0.255 |  |
| 10 (4302) | 0.00413 | 0 | 0 | 0.056 | 0 | 0 | 0 | 0 | 0.259 |  |
| (6516) | 0 | 0 | 0 | 0 | 0 | 5.842 | 0 | 0 | 0.867 | Sorafenib |
| (6498) | 0 | 0 | 0 | 0 | 0 | 0 | 13.087 | 0 | 0.843 | Regorafenib |
| Top-ranked 2-drugs combinations (PDXO-12) | | | | | | | | | | |
| Ranked | Dinaciclib | Carfilzomib | Ixazomib | Omipalisib | Pacritinib | Sorafenib | Regorafenib | UNC0642 | Output |  |
| 1 (4360) | 0.00625 | 0 | 0.02132 | 0 | 0 | 0 | 0 | 0 | 0.336 |  |
| 2 (4727) | 0.00625 | 0 | 0 | 0 | 0 | 0 | 0 | 4.629 | 0.392 | Dinaciclib + UNC0642 |
| 3 (5062) | 0.00625 | 0 | 0 | 0 | 0 | 4.619 | 0 | 0 | 0.447 |  |
| 4 (5194) | 0.00625 | 0 | 0 | 0 | 0 | 0 | 0 | 5.758 | 0.470 | Dinaciclib + UNC0642 |
| 5 (5383) | 0.00625 | 0 | 0 | 0 | 0 | 6.232 | 0 | 0 | 0.504 |  |
| 6 (5449) | 0 | 0.09876 | 0.02132 | 0 | 0 | 0 | 0 | 0 | 0.516 |  |
| 7 (5532) | 0.00625 | 0 | 0.01296 | 0 | 0 | 0 | 0 | 0 | 0.535 |  |
| 8 (5681) | 0 | 0.09876 | 0 | 0 | 0 | 0 | 0 | 4.629 | 0.573 |  |
| 9 (5865) | 0.00625 | 0.09876 | 0 | 0 | 0 | 0 | 0 | 0 | 0.622 |  |
| 10 (5883) | 0.00625 | 0 | 0 | 0 | 0 | 0 | 8.273 | 0 | 0.627 |  |
| (6527) | 0 | 0 | 0 | 0 | 0 | 6.232 | 0 | 0 | 1.101 | Sorafenib |
| (6558) | 0 | 0 | 0 | 0 | 0 | 0 | 11.155 | 0 | 1.404 | Regorafenib |
